# *SORL1* Is Genetically Associated with Late-Onset Alzheimer’s Disease in Japanese, Koreans and Caucasians

**DOI:** 10.1371/journal.pone.0058618

**Published:** 2013-04-02

**Authors:** Akinori Miyashita, Asako Koike, Gyungah Jun, Li-San Wang, Satoshi Takahashi, Etsuro Matsubara, Takeshi Kawarabayashi, Mikio Shoji, Naoki Tomita, Hiroyuki Arai, Takashi Asada, Yasuo Harigaya, Masaki Ikeda, Masakuni Amari, Haruo Hanyu, Susumu Higuchi, Takeshi Ikeuchi, Masatoyo Nishizawa, Masaichi Suga, Yasuhiro Kawase, Hiroyasu Akatsu, Kenji Kosaka, Takayuki Yamamoto, Masaki Imagawa, Tsuyoshi Hamaguchi, Masahito Yamada, Takashi Moriaha, Masatoshi Takeda, Takeo Takao, Kenji Nakata, Yoshikatsu Fujisawa, Ken Sasaki, Ken Watanabe, Kenji Nakashima, Katsuya Urakami, Terumi Ooya, Mitsuo Takahashi, Takefumi Yuzuriha, Kayoko Serikawa, Seishi Yoshimoto, Ryuji Nakagawa, Jong-Won Kim, Chang-Seok Ki, Hong-Hee Won, Duk L. Na, Sang Won Seo, Inhee Mook-Jung, The Alzheimer Disease Genetics Consortium, Peter St. George-Hyslop, Richard Mayeux, Jonathan L. Haines, Margaret A. Pericak-Vance, Makiko Yoshida, Nao Nishida, Katsushi Tokunaga, Ken Yamamoto, Shoji Tsuji, Ichiro Kanazawa, Yasuo Ihara, Gerard D. Schellenberg, Lindsay A. Farrer, Ryozo Kuwano

**Affiliations:** 1 Department of Molecular Genetics, Brain Research Institute, Niigata University, Niigata, Japan; 2 Central Research Laboratory, Hitachi Ltd, Tokyo, Japan; 3 Departments of Medicine (Biomedical Genetics), Ophthalmology and Biostatistics, Boston University Schools of Medicine and Public Health, Boston, Massachusetts, United States of America; 4 Department of Pathology and Laboratory Medicine, University of Pennsylvania School of Medicine, Philadelphia, Pennsylvania, United States of America; 5 Department of Neurology, Iwate Medical University, Morioka, Japan; 6 Department of Neurology, Hirosaki University Graduate School of Medicine, Hirosaki, Japan; 7 Department of Geriatric and Complementary Medicine, Tohoku University Graduate School of Medicine, Sendai, Japan; 8 Department of Psychiatry, University of Tsukuba, Tsukuba, Japan; 9 Department of Neurology, Maebashi Red Cross Hospital, Maebashi, Japan; 10 Department of Neurology, Gunma University Graduate School of Medicine, Maebashi, Japan; 11 Department of Geriatric Medicine, Tokyo Medical University, Tokyo, Japan; 12 Division of Clinical Research, Kurihama Alcoholism Center, Yokosuka, Japan; 13 Department of Neurology, Brain Research Institute, Niigata University, Niigata, Japan; 14 Higashi Niigata Hospital, Niigata, Japan; 15 Kawase Neurology Clinic, Sanjo, Japan; 16 Choju Medical Institute, Fukushimura Hospital, Toyohashi, Japan; 17 Imagawa Clinic, Osaka, Japan; 18 Department of Neurology and Neurobiology of Aging, Kanazawa University Graduate School of Medical Science, Kanazawa, Japan; 19 Department of Psychiatry, Osaka University Graduate School of Medicine, Osaka University, Osaka, Japan; 20 Kurashiki Heisei Hospital, Kurashiki, Japan; 21 Kinoko Espoir Hospital, Kasaoka, Japan; 22 Watanabe Hospital, Tottori, Japan; 23 Department of Neurology Tottori University, Yonago, Japan; 24 Department of Biological Regulation, Section of Environment and Health Science, Tottori University, Yonago, Japan; 25 Town Office, Onan, Japan; 26 Department of Clinical Pharmacology, Fukuoka University, Fukuoka, Japan; 27 Department of Psychiatry, National Hospital Organization, Hizen Psychiatric Center, Saga, Japan; 28 Ureshino-Onsen Hospital, Saga, Japan; 29 Department of Laboratory Medicine & Genetics, Samsung Medical Center, Sungkyunkwan University School of Medicine, Seoul, Korea; 30 Department of Neurology, Samsung Medical Center, Sungkyunkwan University School of Medicine, Seoul, Korea; 31 Department of Biochemistry & Biomedical Sciences, Seoul National University College of Medicine, Seoul, Korea; 32 Tanz Centre for Research in Neurodegenerative Diseases, University of Toronto, Toronto, Canada, and the Department of Clinical Neurosciences, Cambridge Institute for Medical Research, Cambridge, United Kingdom; 33 Taub Institute on Alzheimer's Disease and the Aging Brain, Department of Neurology, Columbia University, New York, United States of America; 34 Department of Molecular Physiology and Biophysics, Vanderbilt University, Nashville, Tennessee, United States of America; 35 The John P. Hussman Institute for Human Genomics, University of Miami, Miami, Florida, United States of America; 36 Department of Human Genetics, University of Tokyo, Tokyo, Japan; 37 Department of Molecular Genetics, Medical Institute of Bioregulation, Kyushu University, Fukuoka, Japan; 38 Department of Neurology, University of Tokyo, Tokyo, Japan; 39 National Center for Neurology and Psychiatry, Kodaira, Japan; 40 Department of Neuropathology, Doshisha University, Kyoto, Japan; 41 Departments of Neurology, Ophthalmology, Genetics & Genomics, and Epidemiology, Boston University Schools of Medicine and Public Health, Boston, Massachusetts, United States of America; Oslo University Hospital, Norway

## Abstract

To discover susceptibility genes of late-onset Alzheimer’s disease (LOAD), we conducted a 3-stage genome-wide association study (GWAS) using three populations: Japanese from the Japanese Genetic Consortium for Alzheimer Disease (JGSCAD), Koreans, and Caucasians from the Alzheimer Disease Genetic Consortium (ADGC). In Stage 1, we evaluated data for 5,877,918 genotyped and imputed SNPs in Japanese cases (n = 1,008) and controls (n = 1,016). Genome-wide significance was observed with 12 SNPs in the *APOE* region. Seven SNPs from other distinct regions with p-values <2×10^−5^ were genotyped in a second Japanese sample (885 cases, 985 controls), and evidence of association was confirmed for one *SORL1* SNP (rs3781834, P = 7.33×10^−7^ in the combined sample). Subsequent analysis combining results for several SORL1 SNPs in the Japanese, Korean (339 cases, 1,129 controls) and Caucasians (11,840 AD cases, 10,931 controls) revealed genome wide significance with rs11218343 (P = 1.77×10^−9^) and rs3781834 (P = 1.04×10^−8^). SNPs in previously established AD loci in Caucasians showed strong evidence of association in Japanese including rs3851179 near *PICALM* (P = 1.71×10^−5^) and rs744373 near *BIN1* (P = 1.39×10^−4^). The associated allele for each of these SNPs was the same as in Caucasians. These data demonstrate for the first time genome-wide significance of LOAD with *SORL1* and confirm the role of other known loci for LOAD in Japanese. Our study highlights the importance of examining associations in multiple ethnic populations.

## Introduction

Alzheimer’s disease (AD) is a progressive neurodegenerative disorder characterized by cognitive dysfunction and memory loss. Multiple rare mutations in *APP*, *PSEN1*, *PSEN2* and *SORL1* account for most cases of early-onset autosomal dominant AD [1], [2]. Risk of late-onset AD (LOAD), the most common type of dementia in the elderly, is associated with complex interactions between genetic and environmental factors. Until recently, *APOE* was the only unequivocally recognized major susceptibility gene for LOAD [1], [3]. Several genome-wide association studies (GWAS) each including more than 5,000 Caucasians identified genome-wide significant associations for LOAD with nine other loci including *ABCA7*, *BIN1*, *CD2AP*, *CD33*, *CLU*, *CR1, EPHA1*, *MS4A* gene cluster, and *PICALM* [4], [5]. To our knowledge, no large GWAS for LOAD has been performed in any Asian population. Because there is a possibility that there exist ethnic-specific LOAD susceptibility variants, we carried out a large-scale GWAS to confirm associations at known loci and identify novel loci for LOAD using a three-stage design including a discovery Japanese cohort and replication cohorts of Japanese, Korean and Caucasian subjects.

## Methods

### Subjects

#### Japanese datasets

Clinically defined subjects were recruited by the Japanese Genetic Study Consortium of Alzheimer’s Disease (JGSCAD: principal investigator, Y.I.) [6], [7]. Probable AD cases were ascertained on the basis of the criteria of the National Institute of Neurological and Communicative Disorders, and Stroke-Alzheimer’s Disease and Related Disorders (NINCDS/ADRDA) [8]. The Mini-Mental State Examination [9], Clinical Dementia Rating [10], and/or Function Assessment Staging [11] were primarily used for evaluation of cognitive impairment. Elders living in an unassisted manner in the local community with no signs of dementia were used as controls. DNA was extracted from peripheral blood leukocytes using standard protocols [6]. For the purpose of this study, the Stage 1 genome-wide association study (GWAS) dataset included 2024 subjects (1008 AD cases and 1016 controls) and the Stage 2 dataset included 1870 subjects (885 AD cases and 985 controls).

#### Korean dataset

A total of 339 subjects with AD were recruited at the Samsung Medical Center in Seoul, Korea. All AD subjects fulfilled NINCDS-ADRDA criteria for probable AD [8]. These subjects underwent a clinical interview and neurological examination that were previously described [12]. The absence of secondary causes of cognitive deficits was assessed by laboratory tests including complete blood count, blood chemistry, vitamin B12/folate, syphilis serology, and thyroid function tests. Conventional brain MRI scans (T1-weighted, T2-weighted, and FLAIR images) confirmed the absence of territorial cerebral infarctions, brain tumors, and other structural lesions. Healthy control subjects (n = 1,129) ages 55 to 85 years were recruited from routine health examination at the same location and showed no evidence of cognitive dysfunction.

#### Alzheimer Disease Genetics Consortium dataset

Summarized information from tests of genetic association of AD with SNPs located in the candidate gene regions was culled from a recent large GWAS conducted by the Alzheimer Disease Genetics Consortium (ADGC) [5]. Results were computed for SNPs throughout the genome in a sample composed of 11,840 AD cases and 10,931 cognitively normal elders from 15 independent Caucasian data sets. Details of the quality control and statistical analysis procedures and genetic models has been published elsewhere [5].

This study was approved by the Boston University Institutional Review Board, Institutional Review Board of Niigata University, and the Institutional Review Boards of all participating institutions. Written informed consent was obtained from all participants. Next of kin, carer takers or guardians consented on the behalf of participants whose capacity to consent was compromised. All subjects were anonymously genotyped. The basic demographics of the cases and controls before QC in each dataset are presented in Table 1.

**Table 1:** Sample size and characteristics of the discovery and replication datasets.

Population (Stage)	Total	Alzheimer Disease Cases					Cognitively Normal Controls			
		N	Female (%)	Age at onset (mean ± SD)	Age at exam (mean ± SD)	*APOE* ε2/ε3/ε4 Frequency	N	Female (%)	Age at exam (mean ± SD)	*APOE* ε2/ε3/ε4 Frequency
Japanese Discovery (Stage 1)	2,024	1,008	723 (72%)	73.0 (4.28)	NA	0.02/0.65/0.33	1,016	583 (57%)	77.0 (5.89)	0.04/0.87/0.09
Japanese Replication (Stage 2)	1,870	885	574 (65%)	74.3 (6.98)	NA	0.02/0.69/0.29	985	618 (63%)	73.74 (5.84)	0.05/0.86/0.09
Korean (Stage 3)	1,468	339	245 (72%)	NA	73.67 (9.49)	0.03/0.70/0.27	1,129	550 (49%)	71.04 (4.86)	0.06/0.85/0.09
Caucasian (Stage 3)	22,771	11,840	7168 (61%)	76.37 (5.18)	80.59 (4.92)	0.04/0.61/0.36	10,931	6418 (59%)	76.77 (3.55)	0.08/0.78/0.14
**TOTAL**	**28,133**	**14,072**					**14,061**			

### Genotyping

GWAS genotyping was performed in the Stage 1 sample using Affymetrix GeneChip 6.0 microarrays containing 909,622 SNPs. Applied Biosystems’ (ABI) TaqMan Assays were used to genotype individual SNPs in the Japanese and Korean replication cohorts. *APOE* genotypes in the Japanese and Korean samples were determined by haplotypes derived from rs7412 and rs429358 which were genotyped using TaqMan Assays. Details of *APOE* genotyping in each ADGC dataset were described previously [13].

### Quality Control and Population Substructure

In the Stage 1 sample, SNPs with a genotype call rate (GCR) <95%, a minor allele frequency (MAF) <0.05, or significant deviation from the Hardy-Weinberg equilibrium (HWE) in controls (*P*<10^−6^) were excluded. After excluding 83,673 low quality and 298,304 low frequency SNPs, we removed 196 subjects with a GCR <95% and 41 subjects whose gender as determined by analysis of X-chromosome data using the PLINK program (ver. 1.06) [14] was inconsistent with the reported gender. The same QC procedures were applied to the Japanese and Korean replication datasets. We examined population substructure in the GWAS dataset by analyzing tagging SNPs from the genome-wide panels using the *smartpca* module from EIGENSTRAT software [15] in a manner described previously [5]. Subsequently, we excluded three subjects who were cryptically related to other subjects in the dataset and 49 individuals who were population outliers. The strength of association of the top 10 principal components (PCs) was tested with AD status. The first three PCs were nominally associated with AD status. A total of 574,828 SNPs and 1,735 subjects comprising 891 cases and 844 controls passed the QC and were used for imputation and in further statistical analyses.

### Genotype Imputation

Genotypes for all SNPs in Japanese and Caucasians were imputed with the Markov Chain haplotyping (MaCH) software [16] using reference haplotypes in the 1000 Genomes database (version released in February 2012 for Japanese datasets and version released in December 2010 for Caucasian datasets). This procedure also filled in missing data for the genotyped SNPs. Imputation quality was determined as *R^2^*, which estimates the squared correlation between imputed and true genotypes. We applied threshold criteria for quality control assessment of imputed SNPs (*R^2^* ≥0.8) as recommended for 1000 Genomes imputed data using the IMPUTE2 program [17]. Genotype probabilities for 5,877,918 genotyped and reliably imputed SNPs with a minor allele frequency (MAF) >0.02 were included in the Japanese GWAS.

### Statistical analysis

Genotyped and imputed SNPs were tested for association with AD in the Stage 1 dataset using a logistic generalized linear model (GLM) controlling for age-at-onset (cases)/age-at-exam (controls), sex and the first three principal components from analysis of of population substructure. Stage 1 analyses were also performed based on a model adjusting for these covariates and the number of *APOE* ε4 alleles. SNPs in the *APOE* region (between map positions 45,000 kb and 45,800 kb on chromosome 19) were also tested for association in ε3/ε3 and ε3/ε4 subgroups. Genotyped SNPs were coded as 0, 1, or 2 according to the number of minor alleles under the additive genetic model. For imputed SNPs, a quantitative estimate between 0 and 2 for the dose of the minor allele were used to incorporate the uncertainty of the imputation estimates. All analyses were performed using PLINK. SNPs attaining a *P* value below 5×10^−5^ were considered for replication in Stage 2. Initially, only one SNP per region was tested in the replication sample to minimize the penalty for multiple testing. Additional SNPs from regions meeting the signifcance threshold in the replication sample were also evaluated. SNPs with a *P* value below 1×10^−5^ in the combined Stages 1 and 2 samples and nominally significant in Stage 2 (*P*<0.05) were advanced to Stage 3.

SNP association results obtained from individual datasets were combined by meta-analysis using the inverse variance method implemented in the software package METAL (http://www.sph.umich.edu/csg/abecasis/Metal/index.html) [18]. An additive model was assumed and the association results across datasets were combined by summing the regression coefficients weighted by the inverse variance of the coefficients. The meta-analysis *P-*value of the association was estimated by the summarized test statistic, after applying a genomic control within each individual study. Effect sizes were weighted by their inverse variance and a combined estimate was calculated by summing the weighted estimates and dividing by the summed weights.

## Results

The quantile-quantile plot indicated limited genomic inflation (λ = 1.04in the Stage 1 GWAS results (Fig. S1). A total of 125 SNPs from seven distinct regions showed evidence of association with *P*<10^−4^ (Table S1, Fig. S2). In addition to *APOE* SNP rs429358 (*P* = 2.46×10^−49^, OR [95% CI]  = 5.5 [4.4–6.9]), 12 other SNPs in the *APOE* region were associated with LOAD at the genome-wide significance level of *P*<5.0×10^−8^. The two most significant results in this group of SNPs were rs12610605 (*PVRL2*: *P* = 1.38×10^−13^, OR [95% CI]  = 1.8 [1.5–2.0]) and rs62117161 (between *CEACAM16* and *BCL3*: *P* = 3.46×10^−12^, OR [95% CI]  = 0.47 [0.38–0.58]). Since imputation in the *APOE* region using the 1000 Genomes reference panel is unreliable [6], we genotyped nine SNPs from this region, spanning multiple linkage disequilibrium (LD) blocks (Fig. S3) and that were nominally significant in the *APOE* ε3/ε3 subgroup, in the Japanese discovery and replication samples using TaqMan assays (Table S2). Genome-wide significant results were obtained for five of these SNPs, but only the association with *PPP1R37* SNP rs 17643262 remained nominally significant after adjustment for the number of *APOE* ε4 alleles (*P* = 3.96×10^−4^) or in analyses stratified by *APOE* genotype (ε3/ε3: *P* = 0.01; ε3/ε4: P = 0.0016).

SNPs from six other distinct chromosomal regions met Stage 2 follow-up criteria (*P*<5×10^−5^) and the top SNP from each region was genotyped in an independent Japanese sample (Table 2). Two SNPs were nominally significant in the replication sample, however the effect direction for *KIAA0494* SNP rs7519866 differed from the discovery sample. Modest evidence for replication was observed only with *SORL1* SNP rs4598682 (*P*≤0.05). Subsequently, we selected an additional four *SORL1* SNPs (rs3781834, rs2282647, rs17125523, and rs3737529) for testing in the Japanese replication sample that were among the most significant in the basic or extended models in the discovery sample (Table S1) and not in LD with rs4598682 (r^2^<0.2, Figure S4). Two of these SNPs (rs3781834 and rs17125523) were chosen also because they were genotyped in the discovery sample and thus would minimize the effects of potential imputation artifacts in meta-analysis of the two Japanese samples. Highly significant results were obtained for *SORL1* SNPs rs4598682 (*P* = 9.51×10^−6^), rs3781834 (*P* = 7.33×10^−7^), rs17125523 (*P* = 5.51×10^−6^), and rs3737529 (*P* = 4.14×10^−6^) after combining results from the discovery and replication samples (Table S3).

**Table 2:** Top-ranked genome-wide association results in the Japanese discovery (Stage 1) sample (P<2.5×10^−5^) and their replication in Japanese (Stage 2).

SNP	CH:MB	Nearest Gene	MA	MAF	# SNPs	Discovery (Stage 1)		Replication (Stage 2)		Meta-Analysis (Stages 1+2)	
						OR (95% CI)	P	OR (95% CI)	P	OR (95% CI)	P
rs7519866	1:47.0	*KIAA0494*	G	0.37	52	0.71 (0.61–0.83)	9.70×10^−6^	1.15 (1.01–1.32)	0.04	0.90 (0.57–1.44)	0.67
rs913360	9:111.7	*PALM2*	G	0.28	20	1.56 (1.43–1.70)	1.83×10^−7^	1.11 (0.96–1.29)	0.16	1.29 (1.15–1.44)	6.6×10^−6^
rs1273007	10:9.0	*LOC338591*	T	0.27	39	0.68 (0.62–0.74)	3.08×10^−6^	0.95 (0.81–1.10)	0.47	0.81 (0.73–0.91)	2.2×10^−4^
rs10898417	11:85.2	*SYTL2*	G	0.15	2	0.59 (0.53–0.66)	1.17×10^−6^	1.02 (0.85–1.22)	0.83	0.82 (0.71–0.93)	0.003
rs4598682	11:121.1	*SORL1*	G	0.23	11	0.68 (0.57–0.81)	2.25×10^−5^	0.83 (0.68–1.00)	0.05	0.75 (0.66–0.85)	9.5×10^−6^
rs11621843	14:92.2	*RIN3*	G	0.26	19	1.47 (1.35–1.60)	5.19×10^−6^	1.03 (0.88–1.20)	0.72	1.21 (1.08–1.36)	8.1×10^−4^

CH:MB, chromosome:position (in megabasepairs, build 19); MA, minor allele; MAF, minor allele frequency; # SNPs, the number of SNPs for which P≤1×10^−4^ in the discovery (Stage 1) sample; OR, odds ratio; *P* P-value;

Selected SNPs represent the strongest association within each locus.

**Figure 1:**
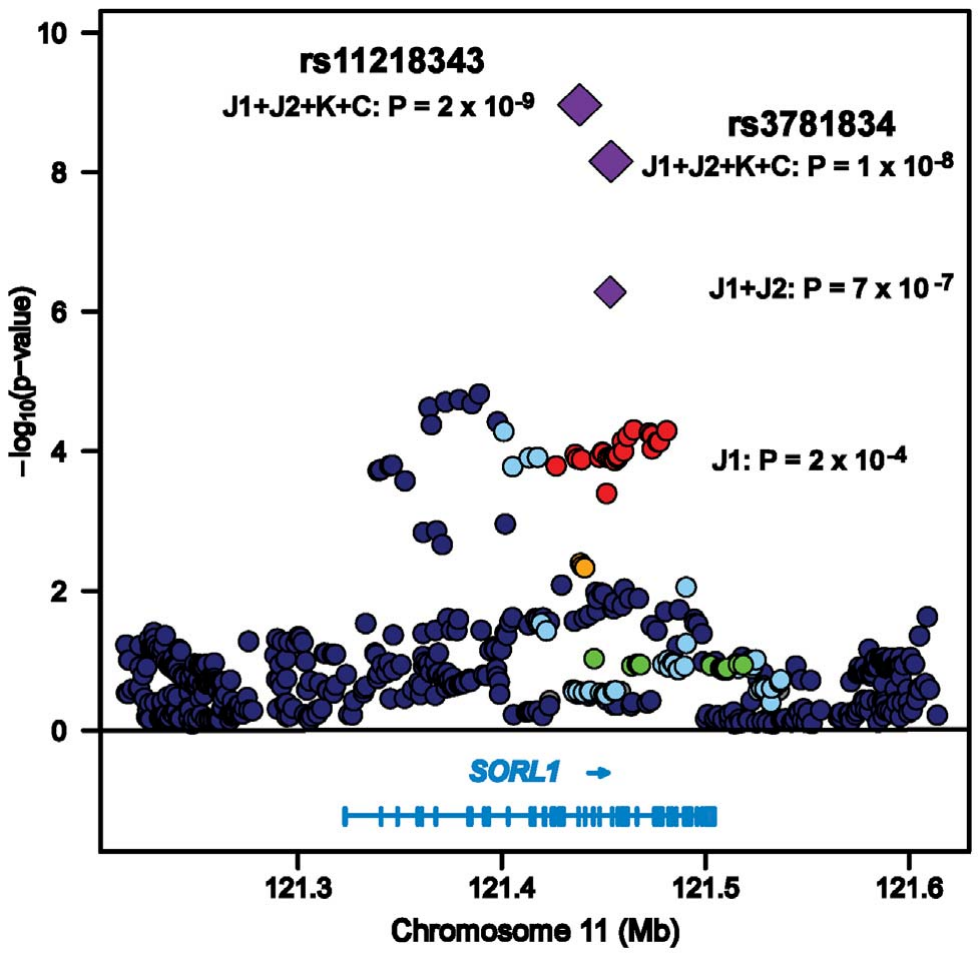
Regional association plot for the *SORL1* region on chromosome 11 in the three-stage design. For each SNP, the chromosomal location is shown on the x-axis and the significance level for association with LOAD is indicated by a –log_10_P value on the y-axis. P-values are expressed as –log_10_(P) (y-axis) for every tested SNP ordered by chromosomal location (x-axis). Genomic position was determined using the NCBI database (Build 37.1). Computed estimates of linkage disequilibrium (LD; r^2^) between SNPs in this region with the top-ranked SNP (rs3781834) in the Japanese discovery (J1) dataset are shown as red circles for r^2^≥0.8, orange circles for 0.5≤r^2^<0.8, light blue circles for 0.2≤r^2^<0.5, and dark blue circles for r^2^<0.2 using hg19/1000 Genomes of Asian populations (ASN; release on November 2010) combined from Han Chinese (CHB) and Japanese (JPT). Meta-analysis *P*-values are shown as purple diamonds for the Japanese datasets (J1+J2) and all datasets (J1+J2+K+C) including Japanese, Korean (K), and Caucasians (C). Two genome-wide significant SNPs in the final stage (rs3781834 and rs11218343) are presented. The gene structure and reading frame are shown below the plot. Exons are denoted with vertical bars. The LD between rs3781834 and rs11218343 is 0.57 in the ASN reference population.

**Figure 2:**
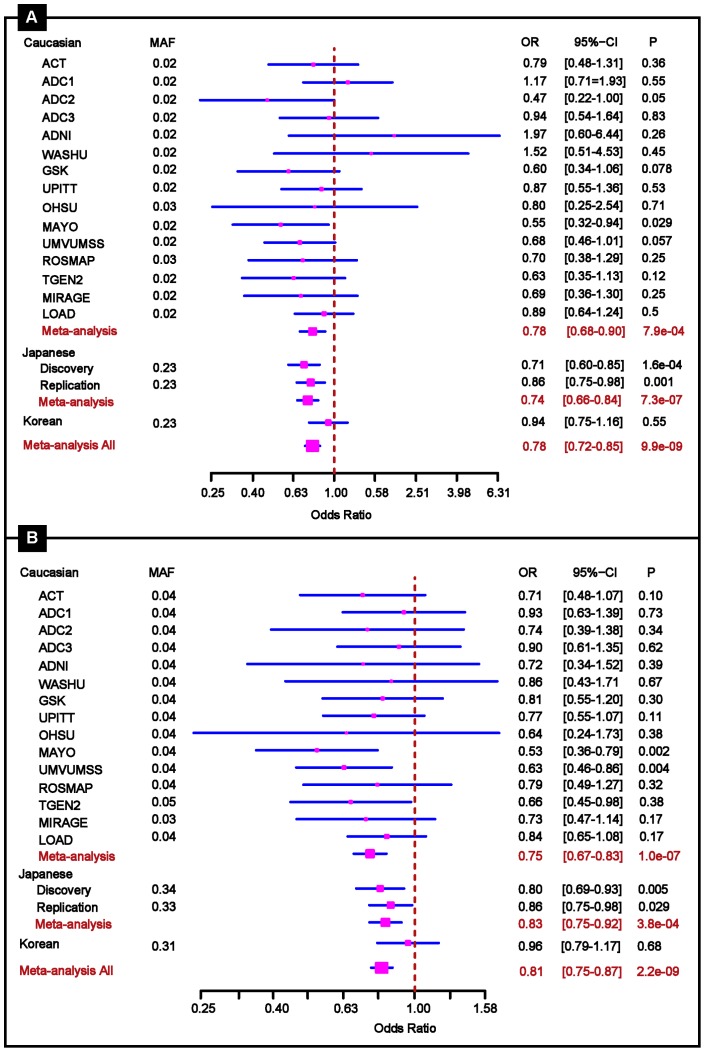
Forest plots of the two most strongly associated SNPs, rs3781834 (A) and rs11218343 (B), in the *SORL1* region showing the strength and pattern of significance in the Japanese discovery and each replication dataset in the model of adjusting for population structure, age, and sex.

These four *SORL1* SNPs showing significant association in the combined samples from Stages 1 and 2 were considered for further replication in Stage 3. We added rs11218343 to this stage of the analysis because it was the most significant *SORL1* SNP in the large Caucasian dataset (*P* = 1.0×10^−7^), a result which emerged after pooling the Caucasian discovery GWAS sample and unpublished data in the replication sample from our previously published GWAS [5]. These five SNPs were subsequently evaluated in Stage 3 by meta analysis including the Stage 1 and 2 Japanese, Korean and ADGC Caucasian datasets. SNPs rs11218343 (*P* = 2.20×10^−9^) and rs3781834 (*P* = 9.90×10^−9^), attained genome-wide significance in the sample of datasets from all stages (Table 3, Fig. 1). There was modest evidence of replication for rs17125523 (meta *P* = 3.30×10^−6^) and rs 3737529 (meta *P* = 5.10×10^−6^). Although the allele frequencies for the top SNPs were very different between the Asian (MAF >0.2) and Caucasian (MAF <0.05) samples (Table 3), there was no evidence of heterogeneity in the magnitude of the odds ratios or effect direction among the population groups (*P*>0.15, Fig. 2). There was no apparent association in the comparably smaller Korean dataset; however, the direction of the effect for each SNP was the same as in the Japanese and Caucasian datsets.

**Table 3:** Meta-analysis of top-ranked association results with *SORL1* in Japanese, Korean, and Caucasian datasets.

SNP	MA	Japanese (Stage 1+2)			Korean (Stage 3)			Caucasian (Stage 3)			Meta-Analysis (Stages 1–3)	
		MAF	OR (95% CI)	P	MAF	OR (95% CI)	P	MAF	OR (95% CI)	P	OR (95% CI)	P
rs4598682	G	0.23	0.75 (0.66–0.85)	9.5×10^−6^		not available		0.02	1.04 (0.85–1.28)	0.68	0.82 (0.72–0.93)	3.6×10^−3^
rs11218343	C	0.34	0.83 (0.75–0.92)	3.8×10^−4^	0.31	0.96 (0.79–1.17)	0.68	0.04	0.75 (0.67–0.83)	1.0×10^−7^	0.81 (0.75–0.87)	2.2×10^−9^
rs3781834	G	0.23	0.74 (0.66–0.84)	7.3×10^−7^	0.23	0.94 (0.75–1.16)	0.55	0.02	0.78 (0.68–0.9)	7.9×10^−4^	0.78 (0.72–0.85)	9.9×10^−9^
rs17125523	G	0.25	0.77 (0.68–0.86)	5.5×10^−6^	0.23	0.96 (0.78–1.19)	0.72	0.02	0.85 (0.74–0.99)	0.034	0.82 (0.76–0.89)	3.3×10^−6^
rs3737529	T	0.25	0.77 (0.68–0.86)	4.1×10^−6^	0.26	1.04 (0.84–1.29)	0.70	0.02	0.83 (0.71–0.97)	0.016	0.82 (0.76–0.89)	5.1×10^−6^

CH:MB, chromosome:position (in megabase pairs, build 19); MA, minor allele; MAF, minor allele frequenc; OR, odds ratio; *P* P-value.

Next, we investigated whether robust genetic associations for LOAD reported previously in Caucasians [4], [5] generalize to Japanese. After correcting for 15 tests, SNPs rs3851179 located approximately 90 kb upstream from *PICALM* (*P* = 1.71×10^−5^) and rs744373 located approximately 30 kb upstream from *BIN1* (*P* = 1.39×10^−4^) were significantly associated with LOAD risk in the Japanese Stage 1 dataset (Table 4). Nominally significant associations were also observed for SNPs in *CR1, CLU,* and *ABCA7.* Of the eight SNPs tested in the small Korean sample, nominally signficant results (*P*<0.05) were obtained for one SNP in *CLU* and *PICALM,* each with the same pattern of association and comparable effect size as in Japanese.

**Table 4:** Association of LOAD in Asians with SNPs showing genome-wide significance in Caucasians.

Gene	CH	BP	SNP	MA	Japanese			Korean		
					MAF	P	OR (95% CI)	MAF	P	OR (95% CI)
CR1	1	207,692,049	rs6656401	A	0.04	***9.02E-03***	1.38 (1.08–1.76)	0.04	3.75E–01	1.24 (0.77–1.99)
CR1	1	207,784,968	rs3818361	A	0.39	2.54E–01	0.94 (0.85–1.04)	0.31	4.08E–01	0.92 (0.76–1.12)
BIN1	2	127,894,615	rs744373	G	0.33	***1.39E*** **–** ***04***	1.25 (1.11–1.4)	0.36	8.05E–01	0.98 (0.81–1.18)
CD2AP	6	47,453,378	rs9349407	G	0.14	3.83E–01	0.94 (0.82–1.08)	NT	–	–
EPHA1	7	143,109,139	rs11767557	C	0.17	6.47E–01	1.03 (0.9–1.17)	NT	–	–
CLU	8	27,456,253	rs2279590	T	0.25	***7.01E*** **–** ***03***	0.85 (0.76–0.96)	0.2	9.70E–02	0.82 (0.65–1.04)
CLU	8	27,464,519	rs11136000	T	0.28	***1.09E*** **–** ***02***	0.87 (0.78–0.97)	0.23	***3.61E*** **–** ***02***	0.79 (0.63–0.98)
CLU	8	27,468,862	rs9331888	G	0.41	6.97E–02	1.1 (0.99–1.22)	0.47	1.92E–01	0.89 (0.74–1.06)
MS4A6A	11	59,939,307	rs610932	T	0.3	7.99E–01	0.99 (0.89–1.1)	NT	–	–
MS4A6A	11	59,971,795	rs670139	T	0.4	8.23E–01	0.99 (0.89–1.09)	NT	–	–
MS4A6A	11	60,034,429	rs4938933	C	0.27	3.23E–01	1.06 (0.95–1.18)	NT	–	–
PICALM	11	85,868,640	rs3851179	T	0.39	***1.71E*** **–** ***05***	0.8 (0.73–0.89)	0.34	***1.99E*** **–** ***02***	0.79 (0.66–0.96)
ABCA7	19	1,046,520	rs3764650	G	0.42	***3.66E*** **–** ***02***	1.13 (1.01–1.27)	NT	–	–
EXOC3L2	19	45,708,888	rs597668	C	0.43	***8.23E*** **–** ***03***	0.88 (0.79–0.97)	0.37	7.31E–01	0.97 (0.8–1.17)
CD33	19	51,727,962	rs3865444	A	0.2	4.92E–01	1.04 (0.92–1.18)	NT	–	–

NT not tested; P<0.05 was italized.

## Discussion

Our multi-stage GWAS of LOAD identified for the first-time genome-wide significant association with *SORL1*. Genetic association with *SORL1* was first established in a study focused on genes encoding proteins involved in vacuolar protein sorting [19]. Most, but not all, subsequent studies in Caucsians replicated this finding (summarized in Alzgene database: http://www.alzgene.org/). Confirmatory evidence of association with *SORL1* SNPs has also been reported in comparatively small samples of Chinese and Japanese (reviewed in [20]). These findings are independent of previous candidate gene studies of *SORL1* in Japanese (two subjects in common) and with Caucasians in the Rogaeva et al. study [19] (less than 2% overlap).

The two genome-wide significant *SORL1* SNPs, rs11218343 and rs3781834 are located at chromosome positions 121,435,587 base pairs and 121,445,940 base pairs, respectively, and thus between the two previously reported strongly associated 3-marker haplotypes that extend upstream from rs641120 (121,380,965 base pairs) and downstream from rs1699102 (121,456,962 base pairs) [19]. A recent meta-analysis including more than 30,000 Caucasian and Asian subjects demonstrated that multiple *SORL1* SNPs in distinct regions are associated with AD [20], a finding substantiated in an association study of *SORL1* SNPs with brain MRI traits in LOAD families [21]. Further analysis of our large Caucasian sample suggests that the association peak at rs3781834 is independent of at least one of the two distinct haplotypes previously associated with AD in an independent sample of non-Hispanic Caucasians, Caribbean Hispanics and Israeli-Arabs (Fig. S5) [19], Since all of the SNPs at the association peaks reported in this study and previously are intronic, functional studies are required to determine the identity of pathogenic variants at these locations.

Remarkably, the less frequent alleles at rs11218343 and rs3781834 are protective in both Japanese and Caucasian datasets with very similar odds ratios (range 0.74 to 0.83) despite the fact that these alleles are much rarer in Caucasians (4% and 2%, respectively) than in Japanese (34% and 23%, respectively). The rarity of these SNPs in Caucasians, as well as allelic heterogeneity, may explain why *SORL1* did not previously emerged as a genome-wide significant AD locus in much larger GWAS [4], [5]. Given the discovery sample size, effect size (odds ratio [OR]  = 0.74) and MAF (0.23) of the top *SORL1* SNP (rs3781834) in the Japanese sample, and a significance level of 2×10^−5^ (i.e., threshold for including a SNP in the Stage 2 replication phase), calculation of power *post hoc* using the PAWE-3D program [22] confirmed that the discovery sample had sufficient power (83.7%). By comparison, the Caucasian sample of 22,771 subjects had only 52.8% power to detect association with this SNP at the observed significance level of 7.9×10^−4^ and OR (0.78) and a much lower MAF (0.02) than in Japanese.

The most significant result in the GWAS in Japanese was obtained for *PALM2* SNP rs913360 (*P* = 1.8×10^−7^), but this SNP was not significant in the Japanese replication sample (*P* = 0.16) and the result for the combined Japanese datasets was less significant than in the discovery sample (*P* = 6.6×10^−6^). There was no evidence in the large Caucasian dataset supporting association for rs913360 (*P* = 0.38) or other *PALM2* SNPs.

We obtained evidence in Japanese and Korean populations for association of AD with the same SNPs in the *PICALM* and *BIN1* regions that were identified as genome-wide significant in multiple large GWAS in Caucasians [4], [5]. There are no previously reported association studies of these loci in Japanese. Several small association studies of *PICALM* in comparatively smaller Chinese samples have yielded conflicting results [23]–[25]. We also found nominally significant associations in the Japanese sample for previously associated SNPs in *CR1, CLU,* and *ABCA7.* Lack of asociation with *EPHA1, CD2AP, MS4A6A,* and *CD33* may be due to insufficient power, different linkage disequilibrium structure of these regions than in Caucasisans, locus heterogeneity or intragenic heterogeneity.

In addition, our analyses showed numerous highly significant results for imputed SNPs in the *APOE* region (including *CEACAM/BCL3, PVRL2, TOMM40,* and *LOC284352)* even after adjustment for the dose of the ε4 allele. However, recognizing that the reliability of imputation is poor for SNPs in this region [13], we genotyped 10 of the significant SNPs in the Japanese discovery and replication datasets. Only one of these results, a *PPP1R37* SNP, was nominally significant after adjustment for dose of ε4. Association of AD with this SNP, which is located approximately 225 kb from *APOE*, has not been observed previously. *PVRL2* and *APOE* are located in a genomic region sandwiched between two recombination hotspots [26], where strong association signals for LOAD have been reproducibly detected in Caucasians [1], [5], but dissipate almost completely for all non-*APOE* loci after conditioning on *APOE*, suggesting that no other loci in this region influence LOAD susceptibility [13]. This conclusion is consistent with the observation of moderate linkage disequilibrium between the SNPs determining *APOE* genotype, rs7412 and rs429358 (Fig. S5), SNPs showing genomewide significant evidence for association with LOAD without adjustment for *APOE* genotype, and our prior LOAD association studies with SNPs in this region among Caucasians [13].

SorL1, also known as SorLA and LR11, and APP proteins are co-localized in the endosomal and Golgi compartments [27]. SorL1 through its co-dependent interaction with vps26 regulates the intracellular transport and processing of APP, resulting in reduction of amyloid beta (Aß) peptide production [20], [27], [28]. *SORL1* knock-out mice carrying both pathogenic mutations in the *PSEN1* (exon 9 deletion) and *APP* (Swedish, K595M/N596L) exhibited increased production and accumulation of Aß [29]. *SORL1* variants might influence the CSF Aß42 level in AD patients [30]. Recently, Pottier et al. sequenced the exomes of 29 index cases with autosomal dominant early-onset AD who lacked mutations in *APP, PSEN1* and *PSEN2* [2]. Seven of these subjects had private *SORL1* mutations (2 nonsense and 2 missense) that were predicted to have a pathogenic effect. By comparison, the two genome-wide significant SNPs in this study are both intronic. It is expected that future large resequencing studies of *SORL1* will identify the functional variants, thus providing important clues about the mechanisms governing normal and abnormal action of SorL1 on processes leading to LOAD. The emergence of *SORL1* as a genome-wide significant locus for AD confirms existing genetic and functional evidence and elevates the importance of intracellular trafficking involving retromer and the Golgi-to- endosome as a key pathway leading to AD [31], [32].

## Supporting Information

Figure S1 **Quantile-quantile (Q-Q) plot of observed (y-axis) vs. expected (x-axis)** ***P*** **-values from tests of association genome-wide (5,877,918 SNPs) adjusted for population structure, age and sex for LOAD in the Japanese discovery sample.** Genomic inflation was low (λ = 1.047).
(TIF)

Figure S2 **Manhattan plot of observed –log_10_** ***P*** **-values for genome-wide SNP association tests for LOAD (y-axis) according to chromosomal location (x-axis) in the Japanese discovery sample adjusted for population structure, age, and sex.** All genome-wide significant SNPs (above the horizontal line corresponding to *P = *5×10^−8^ on the y-axis) are located in the *APOE* region on chromosome 19.
(TIF)

Figure S3 **Linkage disequilibrium (r^2^) among SNPs in the** ***APOE*** **region genotyped using TaqMan calculated in the Japanese discovery (A) and replication (B) datasets.** *APOE* genotype is derived from haplotypes of coding SNPs rs429358 and rs7412.
(TIF)

Figure S4 **Linkage disequilibrium (r^2^) among SNPs in the** ***SORL1*** **region genotyped in the Japanese discovery (A) and replication (B) datasets.**
(TIF)

Figure S5 **Comparison of** ***SORL1*** **association findings in the current study with association signals previously identified by Rogaeva et al.** [**20**] **.** (**A**) Regional association plot of the *SORL1* region. P-values are expressed as –log_10_(P) (y-axis) for every tested SNP ordered by chromosomal location (x-axis) and represented as blue rectangles for the Japanese discovery set (J1), light blue diamonds for the ADGC Caucasian set (C), pink circles for meta-analysis of Japanese discovery and Caucasian sets (J1+C), and red circles for meta-analysis of Japanese discovery, Japanese replication (J2), Korean (K), and Caucasian sets (J1+J2+K+C). The numbers below the line showing the orientation of *SORL1* are the designations for associated SNPs in the Rogaeva et al. study: 8 = rs668387, 9 = rs689021, 10 = rs641120, 11 = rs4935775, 19 = rs2070045, 22 = rs1699102, 23 = rs3824968, 24 = rs2282649, and 25 = rs1010159. Recombination hotspots are indicated by the continuous blue line behind the symbols for the SNP P-values. (**B**) Linkage disequilibrium (r^2^) of the previously associated SNPs in the *SORL1* region [20] in the HapMap 2 reference Japanese population (JPT). The association signal with rs3781834 (contained in Block 2) appears to be independent of one of the distinct AD-associated haplotypes reported by Rogaeva et al. [20] (including SNPs in Block 1), but not necessarily independent of the other AD-associated haplotype reported by Rogaeva et al which includes rs1699102 in Block 2 and the SNPs in Block 3.
(TIF)

Table S1 **Top-ranked GWAS results in the Japanese GWAS dataset (P<1×10** ^−**4**^ **and imputation quality** ≥**0.8) with and withut adjustment for the number of** ***APOE*** **ε4 alleles.**
(DOCX)

Table S2 **Association of individually genotyped SNPs in the** ***APOE*** **region in models with and without adjustment for the number of** ***APOE*** **ε4 alleles.**
(DOCX)

Table S3 **Association results for** ***SORL1*** **SNPs genotyped in the Japanese replication sample.**
(DOCX)
