# Correction: *SORL1* Is Genetically Associated with Late-Onset Alzheimer’s Disease in Japanese, Koreans and Caucasians

**DOI:** 10.1371/annotation/fcb56ea7-d32a-4e45-818d-39cef330c731

**Published:** 2013-07-08

**Authors:** Akinori Miyashita, Asako Koike, Gyungah Jun, Li-San Wang, Satoshi Takahashi, Etsuro Matsubara, Takeshi Kawarabayashi, Mikio Shoji, Naoki Tomita, Hiroyuki Arai, Takashi Asada, Yasuo Harigaya, Masaki Ikeda, Masakuni Amari, Haruo Hanyu, Susumu Higuchi, Takeshi Ikeuchi, Masatoyo Nishizawa, Masaichi Suga, Yasuhiro Kawase, Hiroyasu Akatsu, Kenji Kosaka, Takayuki Yamamoto, Masaki Imagawa, Tsuyoshi Hamaguchi, Masahito Yamada, Takashi Morihara, Masatoshi Takeda, Takeo Takao, Kenji Nakata, Yoshikatsu Fujisawa, Ken Sasaki, Ken Watanabe, Kenji Nakashima, Katsuya Urakami, Terumi Ooya, Mitsuo Takahashi, Takefumi Yuzuriha, Kayoko Serikawa, Seishi Yoshimoto, Ryuji Nakagawa, Jong-Won Kim, Chang-Seok Ki, Hong-Hee Won, Duk L. Na, Sang Won Seo, Inhee Mook-Jung, The Alzheimer Disease Genetics Consortium, Peter St. George-Hyslop, Richard Mayeux, Jonathan L. Haines, Margaret A. Pericak-Vance, Makiko Yoshida, Nao Nishida, Katsushi Tokunaga, Ken Yamamoto, Shoji Tsuji, Ichiro Kanazawa, Yasuo Ihara, Gerard D. Schellenberg, Lindsay A. Farrer, Ryozo Kuwano

The name of the 27th author was spelled incorrectly. The correct name is Takashi Morihara. 

