# Supplementary figures and images for: SORL1 Is Genetically Associated with Late-Onset Alzheimer’s Disease in Japanese, Koreans and Caucasians
Source: PLoS One. 2013 Apr 2;8(4):e58618. doi: 10.1371/journal.pone.0058618 (PMC3614978; doi:10.1371/journal.pone.0058618)

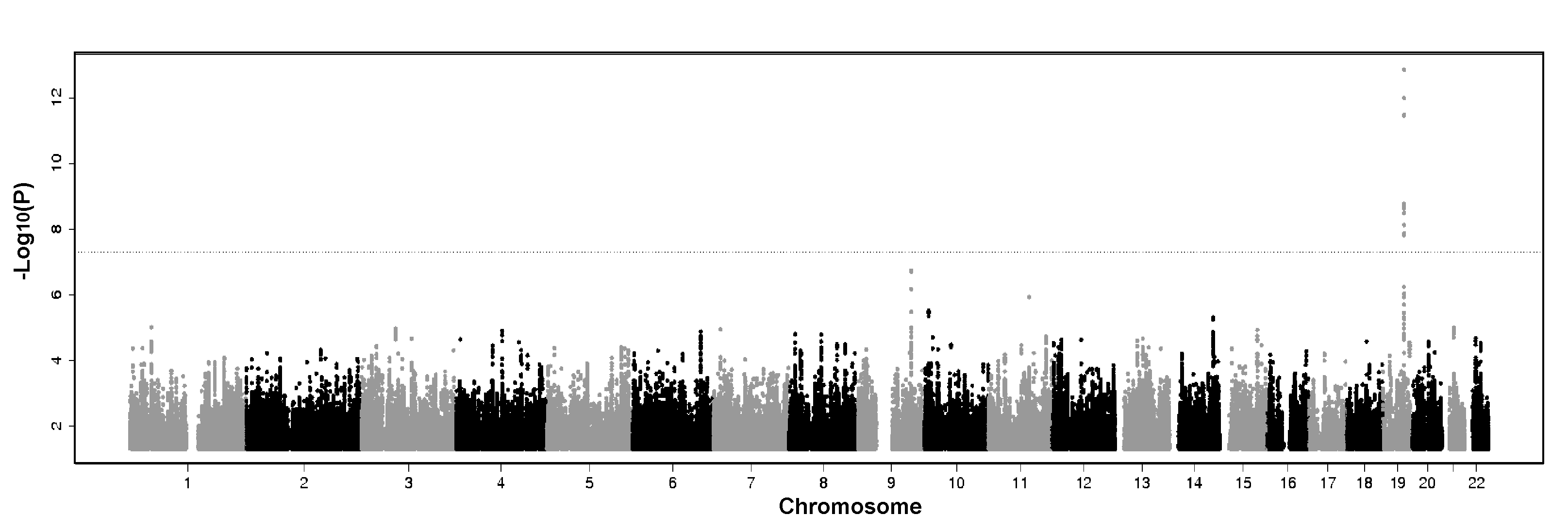

Supplement: Figure S2 — Manhattan plot of observed –log10 P -values for genome-wide SNP association tests for LOAD (y-axis) according to chromosomal location (x-axis) in the Japanese discovery sample adjusted for population structure, age, and sex. All genome-wide significant SNPs (above the horizontal line corresponding to P = 5×10^−8^ on the y-axis) are located in the APOE region on chromosome 19. (TIF) [file pone.0058618.s002.tif]

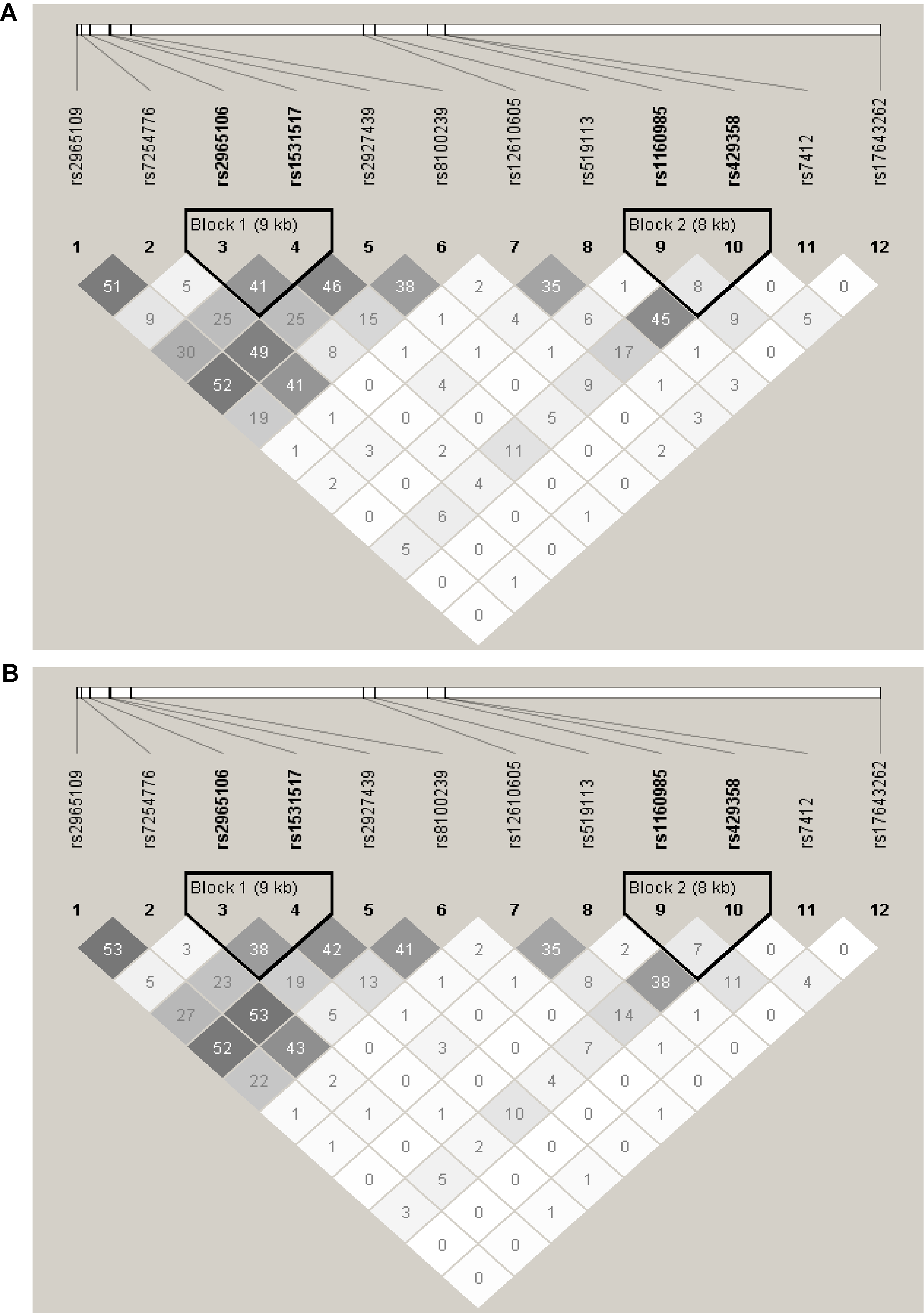

Supplement: Figure S3 — Linkage disequilibrium (r2) among SNPs in the APOE region genotyped using TaqMan calculated in the Japanese discovery (A) and replication (B) datasets. APOE genotype is derived from haplotypes of coding SNPs rs429358 and rs7412. (TIF) [file pone.0058618.s003.tif]

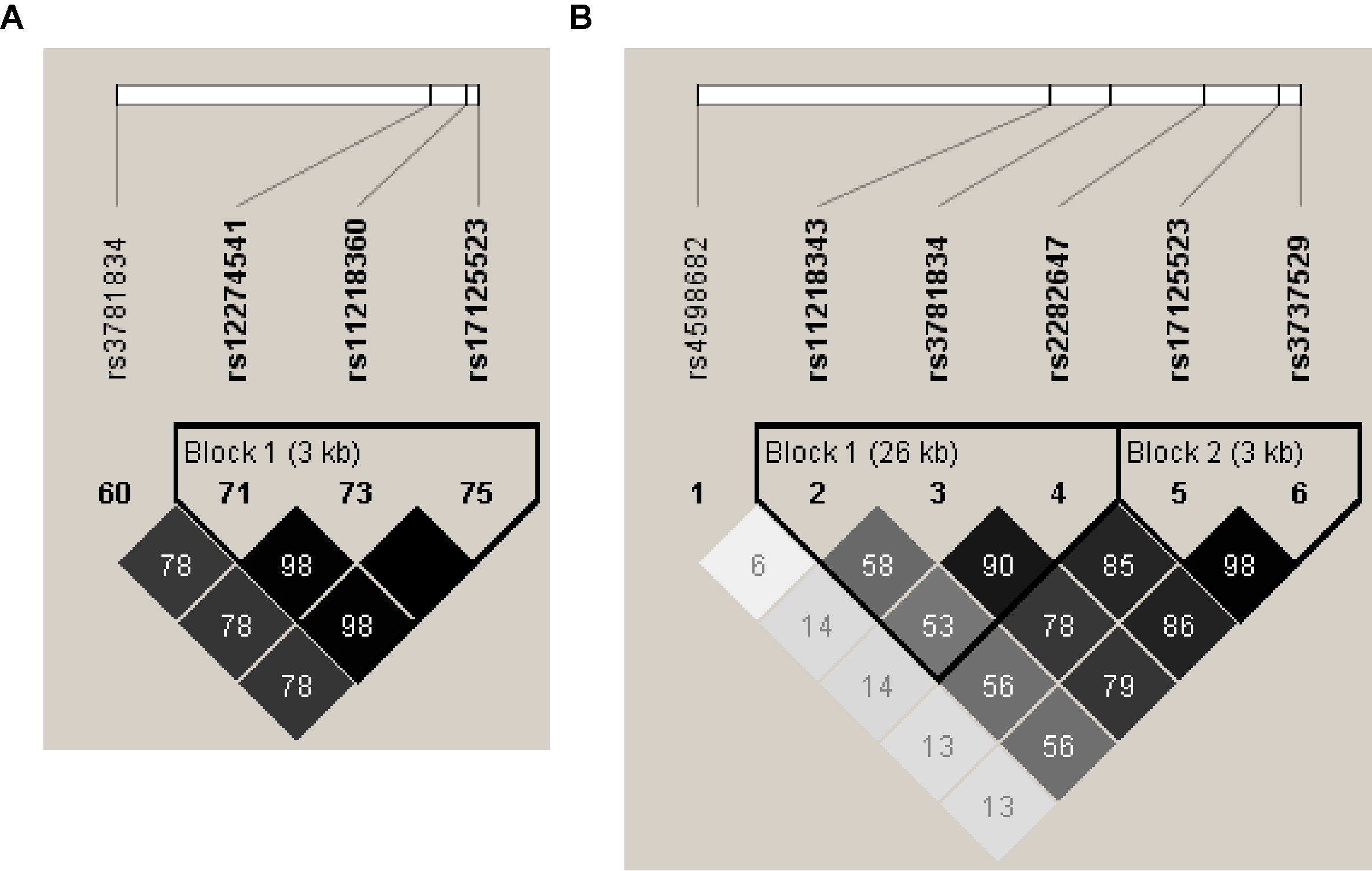

Supplement: Figure S4 — Linkage disequilibrium (r2) among SNPs in the SORL1 region genotyped in the Japanese discovery (A) and replication (B) datasets. (TIF) [file pone.0058618.s004.tif]

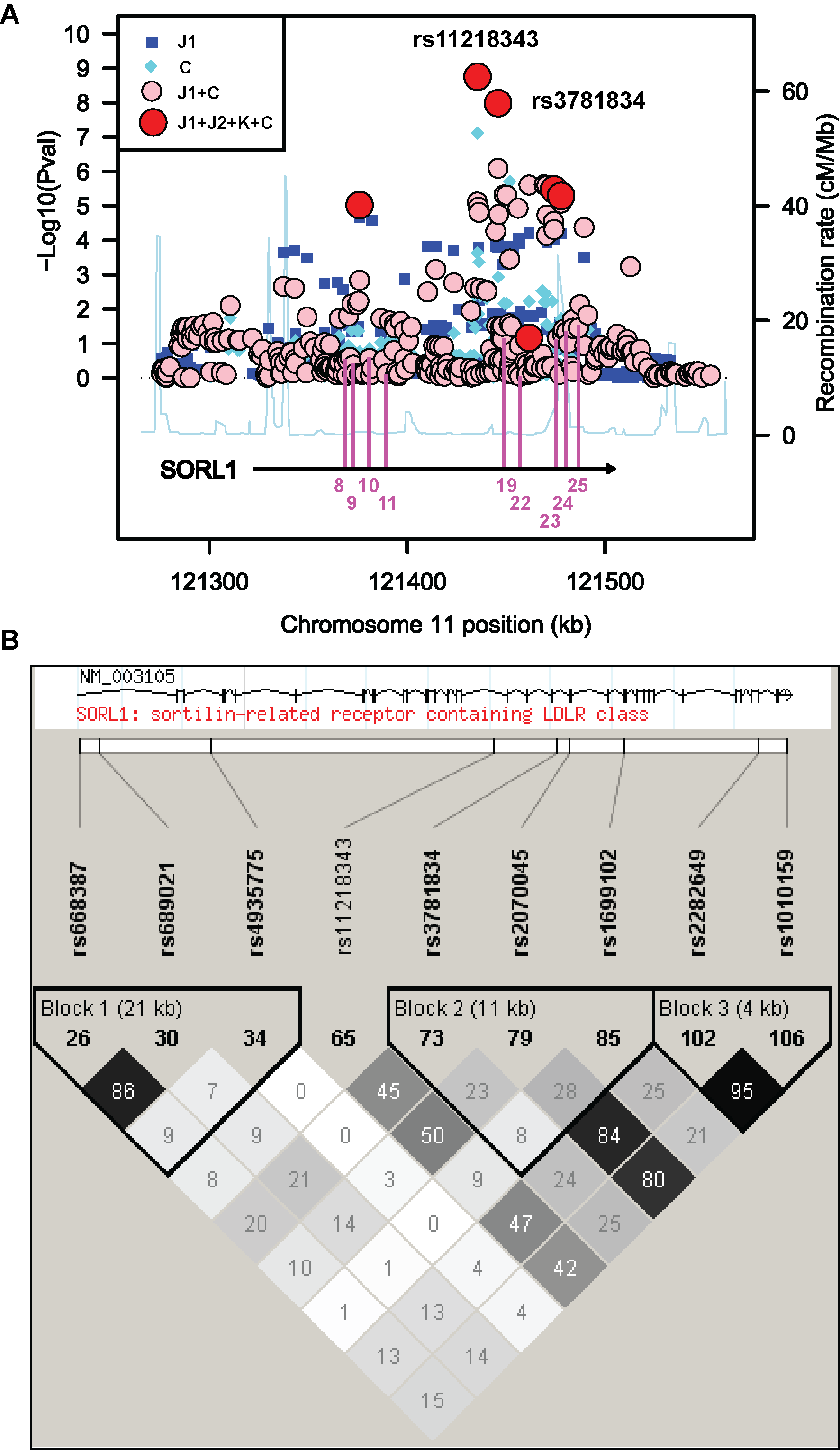

Supplement: Figure S5 — Comparison of SORL1 association findings in the current study with association signals previously identified by Rogaeva et al. [20] . (A) Regional association plot of the SORL1 region. P-values are expressed as –log10(P) (y-axis) for every tested SNP ordered by chromosomal location (x-axis) and represented as blue rectangles for the Japanese discovery set (J1), light blue diamonds for the ADGC Caucasian set (C), pink circles for meta-analysis of Japanese discovery and Caucasian sets (J1+C), and red circles for meta-analysis of Japanese discovery, Japanese replication (J2), Korean (K), and Caucasian sets (J1+J2+K+C). The numbers below the line showing the orientation of SORL1 are the designations for associated SNPs in the Rogaeva et al. study: 8 = rs668387, 9 = rs689021, 10 = rs641120, 11 = rs4935775, 19 = rs2070045, 22 = rs1699102, 23 = rs3824968, 24 = rs2282649, and 25 = rs1010159. Recombination hotspots are indicated by the continuous blue line behind the symbols for the SNP P-values. (B) Linkage disequilibrium (r2) of the previously associated SNPs in the SORL1 region [20] in the HapMap 2 reference Japanese population (JPT). The association signal with rs3781834 (contained in Block 2) appears to be independent of one of the distinct AD-associated haplotypes reported by Rogaeva et al. [20] (including SNPs in Block 1), but not necessarily independent of the other AD-associated haplotype reported by Rogaeva et al which includes rs1699102 in Block 2 and the SNPs in Block 3. (TIF) [file pone.0058618.s005.tif]
