# Supplementary material for: SORL1 Is Genetically Associated with Late-Onset Alzheimer’s Disease in Japanese, Koreans and Caucasians
Source: PLoS One. 2013 Apr 2;8(4):e58618. doi: 10.1371/journal.pone.0058618 (PMC3614978; doi:10.1371/journal.pone.0058618)
Supplement: Table S1 — Top-ranked GWAS results in the Japanese GWAS dataset (P<1×10 −4 and imputation quality ≥0.8) with and withut adjustment for the number of APOE ε4 alleles. (DOCX) [file pone.0058618.s006.docx]

**Supplementary Table 1**. Top-ranked GWAS results in the Japanese GWAS dataset (P<1×10^-4^ and imputation quality > 0.8) with and withut adjustment for the number of APOE ε4 alleles.

|  |  |  |  |  |  |  |  |  | **Basic Model: PCs+Age+Sex** | | **Extended Model: PCs+Age+Sex+APOE** | |
| --- | --- | --- | --- | --- | --- | --- | --- | --- | --- | --- | --- | --- |
| **SNP** | **CH** | **BP** | **Gene** | **A1** | **A2** | **RSQ** | **SNP Type** | **RAF** | **OR (95% CI)** | **P** | **OR (95% CI)** | **P** |
| rs1025806 | 1 | 47,139,103 | ATPAF1 | C | T | 1 | typed | 0.634 | 1.37 (1.18-1.59) | 3.59E-05 | 1.31 (1.12-1.54) | 9.73E-04 |
| rs1048351 | 1 | 47,143,480 | KIAA0494 | C | T | 1 | imputed | 0.635 | 1.37 (1.18-1.59) | 3.08E-05 | 1.31 (1.12-1.54) | 8.72E-04 |
| rs7412469 | 1 | 47,146,533 | KIAA0494 | T | C | 1 | typed | 0.634 | 1.37 (1.18-1.59) | 3.59E-05 | 1.31 (1.12-1.54) | 9.73E-04 |
| rs2304745 | 1 | 47,154,272 | KIAA0494 | C | T | 1 | imputed | 0.634 | 1.37 (1.18-1.59) | 3.60E-05 | 1.31 (1.12-1.54) | 9.76E-04 |
| rs61782563 | 1 | 47,158,266 | KIAA0494 | A | G | 1 | imputed | 0.634 | 1.37 (1.18-1.59) | 3.59E-05 | 1.31 (1.12-1.54) | 9.68E-04 |
| rs61782564 | 1 | 47,158,649 | KIAA0494 | C | T | 1 | imputed | 0.634 | 1.37 (1.18-1.59) | 3.62E-05 | 1.31 (1.12-1.54) | 9.78E-04 |
| rs3766217 | 1 | 47,159,109 | KIAA0494 | G | T | 1 | typed | 0.634 | 1.37 (1.18-1.59) | 3.59E-05 | 1.31 (1.12-1.54) | 9.73E-04 |
| rs34932727 | 1 | 47,161,275 | KIAA0494 | G | A | 0.99 | imputed | 0.631 | 1.37 (1.18-1.59) | 4.38E-05 | 1.31 (1.11-1.53) | 1.13E-03 |
| rs2405026 | 1 | 47,162,648 | KIAA0494 | T | C | 1 | imputed | 0.634 | 1.37 (1.18-1.59) | 3.44E-05 | 1.31 (1.12-1.54) | 9.26E-04 |
| rs4274009 | 1 | 47,165,879 | KIAA0494 | G | A | 1 | imputed | 0.635 | 1.37 (1.18-1.59) | 3.26E-05 | 1.31 (1.12-1.54) | 8.75E-04 |
| rs55914029 | 1 | 47,166,848 | KIAA0494 | G | A | 1 | imputed | 0.635 | 1.37 (1.18-1.59) | 3.10E-05 | 1.31 (1.12-1.54) | 8.29E-04 |
| rs1440486 | 1 | 47,167,238 | KIAA0494 | C | T | 1 | imputed | 0.635 | 1.37 (1.18-1.59) | 3.11E-05 | 1.31 (1.12-1.54) | 8.20E-04 |
| rs60675294 | 1 | 47,169,845 | KIAA0494 | C | G | 1 | imputed | 0.635 | 1.37 (1.18-1.59) | 3.07E-05 | 1.32 (1.12-1.54) | 8.04E-04 |
| rs61782568 | 1 | 47,171,101 | KIAA0494 | T | C | 1 | imputed | 0.635 | 1.37 (1.18-1.59) | 3.06E-05 | 1.32 (1.12-1.54) | 8.01E-04 |
| rs6688195 | 1 | 47,178,195 | KIAA0494 | C | T | 1 | imputed | 0.635 | 1.37 (1.18-1.59) | 2.98E-05 | 1.32 (1.12-1.55) | 7.82E-04 |
| rs3766215 | 1 | 47,181,022 | KIAA0494 | C | T | 1 | imputed | 0.635 | 1.37 (1.18-1.60) | 2.94E-05 | 1.32 (1.12-1.55) | 7.60E-04 |
| rs3737731 | 1 | 47,182,206 | KIAA0494 | C | T | 1 | imputed | 0.635 | 1.38 (1.18-1.60) | 2.92E-05 | 1.32 (1.12-1.55) | 7.36E-04 |
| rs8851 | 1 | 47,184,569 | KIAA0494 | G | C | 0.98 | imputed | 0.626 | 1.37 (1.18-1.59) | 3.33E-05 | 1.32 (1.12-1.55) | 7.96E-04 |
| rs17102473 | 1 | 47,185,155 | KIAA0494 | G | A | 0.99 | imputed | 0.634 | 1.38 (1.19-1.60) | 2.65E-05 | 1.32 (1.13-1.56) | 6.41E-04 |
| rs6429608 | 1 | 47,187,036 | KIAA0494 (2 kb 5') | A | G | 0.97 | imputed | 0.622 | 1.40 (1.21-1.63) | 9.80E-06 | 1.35 (1.14-1.58) | 3.26E-04 |
| rs7519866 | 1 | 47,187,255 | KIAA0494 (2 kb 5') | A | G | 0.97 | imputed | 0.624 | 1.40(1.21-1.63) | 9.66E-06 | 1.35 (1.15-1.59) | 2.89E-04 |
| rs1028867 | 9 | 112,528,882 | PALM2 | A | G | 1 | typed | 0.224 | 1.43 (1.20-1.71) | 8.48E-05 | 1.29 (1.06-1.56) | 1.02E-02 |
| rs11794095 | 9 | 112,605,138 | PALM2-AKAP2 | T | C | 0.9 | imputed | 0.642 | 0.71 (0.61-0.84) | 2.97E-05 | 0.77 (0.65-0.91) | 2.58E-03 |
| rs12235555 | 9 | 112,606,312 | PALM2-AKAP2 | A | T | 0.91 | typed | 0.642 | 0.72 (0.61-0.84) | 3.94E-05 | 0.77 (0.65-0.91) | 2.50E-03 |
| rs10980082 | 9 | 112,606,655 | PALM2-AKAP2 | A | G | 0.92 | imputed | 0.617 | 0.71 (0.61-0.83) | 1.89E-05 | 0.76 (0.64-0.90) | 1.15E-03 |
| rs11789107 | 9 | 112,606,832 | PALM2-AKAP2 | A | G | 0.93 | imputed | 0.611 | 0.71 (0.61-0.83) | 1.38E-05 | 0.75 (0.64-0.89) | 7.09E-04 |
| rs13299867 | 9 | 112,607,260 | PALM2-AKAP2 | C | T | 0.92 | imputed | 0.629 | 0.73 (0.62-0.85) | 6.48E-05 | 0.77 (0.65-0.91) | 2.66E-03 |
| rs13300030 | 9 | 112,607,376 | PALM2-AKAP2 | A | G | 0.97 | imputed | 0.669 | 0.69 (0.59-0.81) | 3.32E-06 | 0.73 (0.62-0.87) | 3.25E-04 |
| rs10816895 | 9 | 112,609,522 | PALM2-AKAP2 | A | C | 1 | typed | 0.694 | 0.71 (0.61-0.83) | 1.95E-05 | 0.74 (0.62-0.87) | 4.60E-04 |
| rs913360 | 9 | 112,611,379 | PALM2-AKAP2 | T | G | 0.99 | imputed | 0.727 | 0.64 (0.54-0.76) | 1.83E-07 | 0.68 (0.57-0.81) | 2.27E-05 |
| rs913361 | 9 | 112,611,525 | PALM2-AKAP2 | G | A | 1 | typed | 0.726 | 0.64 (0.55-0.76) | 1.96E-07 | 0.68 (0.57-0.81) | 2.60E-05 |
| rs13283845 | 9 | 112,613,183 | PALM2-AKAP2 | C | T | 0.99 | imputed | 0.672 | 0.67 (0.58-0.79) | 6.82E-07 | 0.71 (0.60-0.84) | 7.64E-05 |
| rs34879704 | 9 | 112,619,327 | PALM2-AKAP2 | G | A | 0.99 | imputed | 0.722 | 0.72 (0.61-0.84) | 6.90E-05 | 0.73 (0.61-0.88) | 6.57E-04 |
| rs13298370 | 9 | 112,627,830 | PALM2-AKAP2 | G | C | 0.98 | typed | 0.656 | 0.71 (0.61-0.83) | 1.02E-05 | 0.74 (0.63-0.88) | 4.16E-04 |
| rs13299632 | 9 | 112,630,042 | PALM2-AKAP2 | A | G | 1 | typed | 0.662 | 0.73 (0.63-0.85) | 4.86E-05 | 0.77 (0.65-0.91) | 1.69E-03 |
| rs895068 | 10 | 8,966,406 | intergenic | G | T | 1 | typed | 0.729 | 1.46 (1.24-1.72) | 4.45E-06 | 1.43 (1.20-1.71) | 5.90E-05 |
| rs1243418 | 10 | 8,966,800 | intergenic | T | G | 1 | typed | 0.729 | 1.47 (1.25-1.73) | 3.37E-06 | 1.44 (1.21-1.72) | 4.70E-05 |
| rs1243417 | 10 | 8,967,379 | intergenic | A | T | 1 | imputed | 0.729 | 1.48 (1.25-1.74) | 3.10E-06 | 1.45 (1.21-1.73) | 4.18E-05 |
| rs1273007 | 10 | 8,967,522 | intergenic | C | T | 1 | imputed | 0.729 | 1.48 (1.25-1.74) | 3.08E-06 | 1.45 (1.21-1.73) | 4.17E-05 |
| rs1243416 | 10 | 8,967,716 | intergenic | G | A | 1 | imputed | 0.729 | 1.48 (1.25-1.74) | 3.09E-06 | 1.45 (1.21-1.73) | 4.18E-05 |
| rs1243413 | 10 | 8,969,083 | intergenic | A | G | 1 | imputed | 0.73 | 1.47 (1.25-1.74) | 3.66E-06 | 1.45 (1.21-1.73) | 4.50E-05 |
| rs10898417 | 11 | 85,547,503 | intergenic | A | G | 1 | typed | 0.872 | 1.68 (1.37-2.08) | 1.17E-06 | 1.70 (1.35-2.13) | 4.51E-06 |
| rs4598682 | 11 | 121,375,951 | SORL1 | A | G | 0.99 | imputed | 0.769 | 1.47 (1.23-1.75) | 2.25E-05 | 1.59 (1.31-1.92) | 3.03E-06 |
| rs6589884 | 11 | 121,382,172 | SORL1 | A | T | 0.97 | imputed | 0.768 | 1.46 (1.23-1.75) | 2.58E-05 | 1.59 (1.31-1.93) | 2.86E-06 |
| rs2282647 | 11 | 121,461,593 | SORL1 | G | C | 0.98 | imputed | 0.777 | 1.44 (1.21-1.73) | 6.12E-05 | 1.55 (1.27-1.88) | 1.52E-05 |
| rs3737529 | 11 | 121,477,816 | SORL1 | C | T | 1 | imputed | 0.753 | 1.42 (1.2-1.68) | 6.30E-05 | 1.52 (1.26-1.83) | 1.51E-05 |
| rs12274536 | 11 | 121,470,620 | SORL1 | C | G | 1 | imputed | 0.752 | 1.41 (1.19-1.68) | 7.36E-05 | 1.51 (1.25-1.82) | 1.98E-05 |
| rs12287339 | 11 | 121,474,025 | SORL1 | T | C | 1 | imputed | 0.752 | 1.41 (1.19-1.67) | 8.93E-05 | 1.5 (1.25-1.82) | 2.19E-05 |
| rs11218360 | 11 | 121,473,391 | SORL1 | T | C | 1 | typed | 0.752 | 1.41 (1.19-1.67) | 9.05E-05 | 1.5 (1.25-1.81) | 2.20E-05 |
| rs17125523 | 11 | 121,474,239 | SORL1 | A | G | 1 | typed | 0.752 | 1.41 (1.19-1.67) | 9.05E-05 | 1.5 (1.25-1.81) | 2.20E-05 |
| rs12274541 | 11 | 121,470,646 | SORL1 | C | T | 1 | typed | 0.751 | 1.40 (1.18-1.66) | 1.14E-04 | 1.5 (1.25-1.81) | 2.16E-05 |
| rs2276345 | 11 | 121,414,456 | SORL1 | A | C | 0.98 | imputed | 0.773 | 1.4 (1.18-1.67) | 1.51E-04 | 1.46 (1.21-1.77) | 9.34E-05 |
| rs17125473 | 11 | 121,444,811 | SORL1 | G | C | 1 | imputed | 0.773 | 1.41 (1.18-1.68) | 1.51E-04 | 1.50 (1.23-1.82) | 4.18E-05 |
| rs59260691 | 11 | 121,450,332 | SORL1 | G | A | 1 | imputed | 0.774 | 1.41 (1.18-1.68) | 1.52E-04 | 1.5 (1.23-1.82) | 4.42E-05 |
| rs3781837 | 11 | 121,448,972 | SORL1 | T | C | 1 | imputed | 0.774 | 1.41 (1.18-1.68) | 1.53E-04 | 1.5 (1.23-1.82) | 4.45E-05 |
| rs58698151 | 11 | 121,453,779 | SORL1 | A | T | 0.99 | imputed | 0.774 | 1.41 (1.18-1.68) | 1.55E-04 | 1.5 (1.23-1.82) | 4.45E-05 |
| rs3781834 | 11 | 121,445,940 | SORL1 | A | G | 1 | typed | 0.773 | 1.40 (1.18-1.67) | 1.58E-04 | 1.50 (1.23-1.82) | 4.27E-05 |
| rs3781831 | 11 | 121,436,004 | SORL1 | G | A | 1 | imputed | 0.772 | 1.40 (1.18-1.67) | 1.63E-04 | 1.49 (1.23-1.81) | 4.86E-05 |
| rs7120354 | 11 | 121,452,354 | SORL1 | G | A | 1 | imputed | 0.773 | 1.40 (1.18-1.68) | 1.68E-04 | 1.5 (1.23-1.82) | 4.94E-05 |
| rs11604897 | 11 | 121,423,552 | SORL1 | C | T | 0.94 | imputed | 0.773 | 1.41 (1.18-1.69) | 2.01E-04 | 1.50 (1.23-1.82) | 6.60E-05 |
| rs12882803 | 14 | 93,091,341 | RIN3 | G | C | 0.99 | imputed | 0.709 | 0.72 (0.61-0.84) | 5.93E-05 | 0.68 (0.57-0.81) | 2.40E-05 |
| rs61975788 | 14 | 93,091,558 | RIN3 | C | T | 0.98 | imputed | 0.711 | 0.72 (0.61-0.84) | 6.36E-05 | 0.68 (0.57-0.82) | 2.57E-05 |
| rs2181377 | 14 | 93,093,155 | RIN3 | G | C | 0.99 | imputed | 0.71 | 0.71 (0.61-0.84) | 5.35E-05 | 0.68 (0.57-0.81) | 2.28E-05 |
| rs11160083 | 14 | 93,093,798 | RIN3 | G | A | 0.97 | imputed | 0.703 | 0.71 (0.60-0.84) | 5.08E-05 | 0.68 (0.57-0.81) | 2.30E-05 |
| rs12590196 | 14 | 93,096,561 | RIN3 | C | T | 0.99 | imputed | 0.71 | 0.71 (0.60-0.84) | 3.90E-05 | 0.68 (0.57-0.81) | 1.81E-05 |
| rs12590180 | 14 | 93,096,616 | RIN3 | A | G | 0.99 | imputed | 0.709 | 0.71 (0.60-0.83) | 2.85E-05 | 0.67 (0.56-0.8) | 1.18E-05 |
| rs12590218 | 14 | 93,096,711 | RIN3 | C | A | 0.99 | imputed | 0.71 | 0.71 (0.60-0.84) | 3.93E-05 | 0.68 (0.57-0.81) | 1.85E-05 |
| rs11621441 | 14 | 93,098,070 | RIN3 | C | T | 0.99 | imputed | 0.712 | 0.71 (0.60-0.83) | 3.44E-05 | 0.68 (0.57-0.81) | 1.57E-05 |
| rs11626270 | 14 | 93,098,452 | RIN3 | T | C | 0.97 | imputed | 0.7 | 0.73 (0.62-0.86) | 1.42E-04 | 0.7 (0.58-0.83) | 5.90E-05 |
| rs11621652 | 14 | 93,098,472 | RIN3 | G | A | 1 | typed | 0.71 | 0.70 (0.60-0.83) | 2.42E-05 | 0.68 (0.57-0.81) | 1.46E-05 |
| rs11160084 | 14 | 93,099,369 | RIN3 | G | A | 1 | imputed | 0.71 | 0.70 (0.60-0.83) | 2.14E-05 | 0.67 (0.56-0.8) | 9.90E-06 |
| rs4904964 | 14 | 93,099,867 | RIN3 | A | C | 1 | typed | 0.69 | 0.73 (0.63-0.86) | 1.14E-04 | 0.7 (0.59-0.83) | 4.53E-05 |
| rs8015224 | 14 | 93,101,074 | RIN3 | T | G | 1 | imputed | 0.69 | 0.73 (0.63-0.86) | 1.20E-04 | 0.7 (0.59-0.83) | 4.60E-05 |
| rs4900138 | 14 | 93,104,025 | RIN3 | G | C | 0.99 | imputed | 0.689 | 0.73 (0.63-0.86) | 1.19E-04 | 0.7 (0.59-0.83) | 4.41E-05 |
| rs11627032 | 14 | 93,104,072 | RIN3 | T | C | 0.99 | imputed | 0.71 | 0.71 (0.60-0.83) | 3.14E-05 | 0.68 (0.57-0.81) | 1.58E-05 |
| rs11623618 | 14 | 93,104,959 | RIN3 | G | A | 0.99 | imputed | 0.709 | 0.71 (0.60-0.83) | 3.19E-05 | 0.68 (0.57-0.81) | 1.46E-05 |
| rs35268629 | 14 | 93,110,372 | RIN3 | G | A | 1 | imputed | 0.709 | 0.71 (0.6-0.83) | 2.57E-05 | 0.67 (0.56-0.8) | 9.49E-06 |
| rs11160085 | 14 | 93,112,102 | RIN3 | T | C | 0.95 | imputed | 0.693 | 0.73 (0.62-0.85) | 1.21E-04 | 0.69 (0.58-0.83) | 4.88E-05 |
| rs7151520 | 14 | 93,113,538 | RIN3 | T | C | 1 | typed | 0.708 | 0.70 (0.60-0.83) | 2.08E-05 | 0.67 (0.56-0.8) | 6.86E-06 |
| rs7151531 | 14 | 93,113,547 | RIN3 | T | C | 1 | imputed | 0.708 | 0.71 (0.60-0.83) | 2.45E-05 | 0.67 (0.56-0.8) | 8.55E-06 |
| rs7150776 | 14 | 93,113,821 | RIN3 | C | T | 1 | imputed | 0.709 | 0.70 (0.60-0.83) | 2.40E-05 | 0.67 (0.56-0.8) | 8.29E-06 |
| rs35326193 | 14 | 93,114,055 | RIN3 | A | T | 0.99 | imputed | 0.709 | 0.70 (0.60-0.83) | 2.23E-05 | 0.67 (0.56-0.8) | 7.82E-06 |
| rs11621843 | 14 | 93,116,124 | RIN3 | A | G | 1 | typed | 0.73 | 0.68 (0.58-0.80) | 5.19E-06 | 0.66 (0.55-0.79) | 5.96E-06 |
| rs3829948 | 14 | 93,116,826 | RIN3 | T | G | 0.99 | imputed | 0.73 | 0.68 (0.58-0.80) | 5.63E-06 | 0.66 (0.55-0.79) | 7.95E-06 |
| rs2402173 | 14 | 93,116,878 | RIN3 | G | C | 0.99 | typed | 0.729 | 0.68 (0.58-0.80) | 5.29E-06 | 0.66 (0.55-0.79) | 7.45E-06 |
| rs3814830 | 14 | 93,118,198 | RIN3 | C | T | 0.97 | imputed | 0.714 | 0.70 (0.59-0.83) | 2.06E-05 | 0.68 (0.57-0.81) | 1.78E-05 |
| rs61992600 | 14 | 93,119,725 | RIN3 | G | A | 0.99 | imputed | 0.719 | 0.68 (0.58-0.80) | 4.89E-06 | 0.66 (0.55-0.79) | 6.77E-06 |
| rs7160920 | 14 | 93,120,620 | RIN3 | C | T | 0.97 | imputed | 0.703 | 0.72 (0.61-0.84) | 6.23E-05 | 0.7 (0.58-0.83) | 4.71E-05 |
| rs11629324 | 14 | 93,120,719 | RIN3 | C | T | 1 | typed | 0.708 | 0.70 (0.60-0.82) | 1.53E-05 | 0.68 (0.57-0.81) | 1.89E-05 |
| rs11849307 | 14 | 93,121,370 | RIN3 | T | C | 1 | imputed | 0.707 | 0.70 (0.60-0.82) | 1.49E-05 | 0.68 (0.57-0.81) | 1.84E-05 |
| rs11624035 | 14 | 93,123,142 | RIN3 | A | G | 1 | imputed | 0.707 | 0.70 (0.60-0.82) | 1.49E-05 | 0.68 (0.57-0.81) | 1.86E-05 |
| rs56019622 | 14 | 93,124,994 | RIN3 | C | G | 1 | imputed | 0.707 | 0.70 (0.59-0.82) | 1.35E-05 | 0.68 (0.57-0.81) | 1.76E-05 |
| rs7155457 | 14 | 93,126,557 | RIN3 | T | C | 1 | typed | 0.707 | 0.70 (0.60-0.82) | 1.39E-05 | 0.68 (0.57-0.81) | 1.67E-05 |
| rs11160087 | 14 | 93,129,982 | RIN3 | G | A | 0.92 | imputed | 0.71 | 0.70 (0.59-0.83) | 2.61E-05 | 0.68 (0.57-0.81) | 2.56E-05 |
| rs4900140 | 14 | 93,130,227 | RIN3 | A | G | 0.87 | imputed | 0.714 | 0.69 (0.58-0.82) | 2.72E-05 | 0.67 (0.55-0.81) | 2.54E-05 |
| rs2965109 | 19 | 45,225,345 | CEACAM16/BCL3 | C | T | 1 | typed | 0.564 | 0.72 (0.62-0.84) | 1.70E-05 | 0.73 (0.62-0.86) | 1.36E-04 |
| rs7254776 | 19 | 45,227,742 | CEACAM16/BCL3 | T | C | 1 | typed | 0.697 | 0.70 (0.59-0.82) | 1.57E-05 | 0.67 (0.56-0.8) | 8.82E-06 |
| rs4803745 | 19 | 45,230,752 | CEACAM16/BCL3 | A | T | 0.89 | imputed | 0.441 | 1.34 (1.14-1.57) | 2.59E-04 | 1.41 (1.19-1.67) | 7.84E-05 |
| rs1551891 | 19 | 45,231,821 | CEACAM16/BCL3 | G | A | 0.9 | imputed | 0.776 | 0.57 (0.48-0.69) | 3.23E-09 | 0.57 (0.47-0.7) | **3.16E-08** |
| rs2965106 | 19 | 45,232,374 | CEACAM16/BCL3 | G | A | 1 | typed | 0.408 | 1.41 (1.22-1.64) | 3.64E-06 | 1.41 (1.2-1.65) | 2.55E-05 |
| rs62117161 | 19 | 45,233,385 | CEACAM16/BCL3 | A | G | 0.89 | imputed | 0.828 | 0.47 (0.38-0.58) | 3.46E-12 | 0.46 (0.36-0.57) | **1.29E-11** |
| rs8104467 | 19 | 45,233,489 | CEACAM16/BCL3 | A | G | 0.92 | imputed | 0.372 | 1.42 (1.22-1.66) | 7.78E-06 | 1.45 (1.22-1.72) | 1.67E-05 |
| rs8108110 | 19 | 45,234,124 | CEACAM16/BCL3 | A | G | 0.88 | imputed | 0.388 | 1.44 (1.23-1.69) | 5.43E-06 | 1.47 (1.24-1.75) | 1.05E-05 |
| rs2965101 | 19 | 45,237,812 | CEACAM16/BCL3 | T | C | 0.92 | imputed | 0.659 | 0.71 (0.60-0.83) | 1.90E-05 | 0.69 (0.58-0.82) | 3.17E-05 |
| rs2927436 | 19 | 45,239,185 | CEACAM16/BCL3 | A | G | 0.95 | imputed | 0.625 | 0.70 (0.60-0.81) | 4.70E-06 | 0.7 (0.59-0.82) | 2.30E-05 |
| rs62117162 | 19 | 45,239,536 | CEACAM16/BCL3 | C | A | 0.9 | imputed | 0.774 | 0.57 (0.47-0.68) | 1.75E-09 | 0.56 (0.46-0.69) | **1.75E-08** |
| rs1531517 | 19 | 45,242,173 | CEACAM16/BCL3 | G | A | 0.88 | imputed | 0.774 | 0.57 (0.47-0.68) | 1.69E-09 | 0.56 (0.46-0.69) | **1.74E-08** |
| rs2927439 | 19 | 45,242,740 | CEACAM16/BCL3 | A | G | 1 | typed | 0.611 | 0.72 (0.62-0.83) | 1.06E-05 | 0.72 (0.61-0.85) | 6.83E-05 |
| rs62117204 | 19 | 45,242,967 | CEACAM16/BCL3 | C | T | 0.85 | imputed | 0.787 | 0.55 (0.46-0.67) | 2.39E-09 | 0.55 (0.45-0.68) | **2.61E-08** |
| rs12610605 | 19 | 45,370,838 | PVRL2 | G | A | 1 | typed | 0.526 | 1.75 (1.51-2.03) | 1.38E-13 | 1.72 (1.46-2.01) | **3.48E-11** |
| rs157584 | 19 | 45,396,899 | TOMM40 | T | C | 0.84 | imputed | 0.737 | 1.55 (1.29-1.85) | 2.00E-06 | 1.5 (1.23-1.82) | 5.54E-05 |
| rs157588 | 19 | 45,398,264 | TOMM40 | C | T | 0.85 | imputed | 0.739 | 1.56 (1.30-1.86) | 1.18E-06 | 1.51 (1.24-1.83) | 3.34E-05 |
| rs157590 | 19 | 45,398,716 | TOMM40 | A | C | 0.86 | imputed | 0.737 | 1.56 (1.31-1.86) | 9.28E-07 | 1.51 (1.25-1.83) | 2.83E-05 |
| rs1160985 | 19 | 45,403,412 | TOMM40 | C | T | 1 | typed | 0.752 | 1.64 (1.39-1.95) | 7.52E-09 | 1.58 (1.32-1.9) | 9.61E-07 |
| rs760136 | 19 | 45,403,858 | TOMM40 | A | G | 0.97 | imputed | 0.755 | 1.64 (1.38-1.94) | 1.52E-08 | 1.59 (1.32-1.91) | 1.13E-06 |
| rs741780 | 19 | 45,404,431 | TOMM40 | T | C | 0.97 | imputed | 0.755 | 1.64 (1.38-1.95) | 1.43E-08 | 1.59 (1.32-1.92) | 1.07E-06 |
| rs1038025 | 19 | 45,404,972 | TOMM40 | T | C | 0.97 | imputed | 0.755 | 1.64 (1.38-1.94) | 1.49E-08 | 1.59 (1.32-1.91) | 1.11E-06 |
| rs1038026 | 19 | 45,405,062 | TOMM40 | A | G | 0.97 | imputed | 0.755 | 1.64 (1.38-1.94) | 1.47E-08 | 1.59 (1.32-1.91) | 1.10E-06 |
| rs7259620 | 19 | 45,407,788 | APOE | G | A | 0.95 | imputed | 0.757 | 1.65 (1.39-1.97) | 1.39E-08 | 1.6 (1.32-1.93) | 1.15E-06 |
| rs10405086 | 19 | 45,627,235 | LOC284352 | C | T | 0.98 | imputed | 0.827 | 0.65 (0.54-0.8) | 2.13E-05 | 0.66 (0.54-0.82) | 1.25E-04 |
| rs10413253 | 19 | 45,628,566 | LOC284352 | C | T | 0.95 | imputed | 0.822 | 0.65 (0.54-0.79) | 2.06E-05 | 0.66 (0.53-0.81) | 1.03E-04 |
| rs17643262 | 19 | 45,631,816 | LOC284352 | G | A | 1 | typed | 0.827 | 0.65 (0.54-0.79) | 1.58E-05 | 0.66 (0.54-0.81) | 9.78E-05 |
| rs754366 | 19 | 45,633,686 | LOC284352 | G | A | 0.97 | imputed | 0.782 | 0.69 (0.58-0.83) | 5.90E-05 | 0.68 (0.56-0.83) | 1.14E-04 |
| rs10408847 | 19 | 45,634,682 | LOC284352 | G | C | 0.98 | imputed | 0.826 | 0.65 (0.54-0.80) | 2.11E-05 | 0.66 (0.54-0.82) | 1.21E-04 |

RAF = referrent allele (A1) frequency ; NC = model did not converge
