# Supplementary material for: SORL1 Is Genetically Associated with Late-Onset Alzheimer’s Disease in Japanese, Koreans and Caucasians
Source: PLoS One. 2013 Apr 2;8(4):e58618. doi: 10.1371/journal.pone.0058618 (PMC3614978; doi:10.1371/journal.pone.0058618)
Supplement: Table S2 — Association of individually genotyped SNPs in the APOE region in models with and without adjustment for the number of APOE ε4 alleles. (DOCX) [file pone.0058618.s007.docx]

**Supplementary Table 2.** Association of individually genotyped SNPs in the APOE region in models with and without adjustment for the number of APOE ε4 alleles.

|  |  |  |  |  |  | | |  | **Unadjusted for APOE ε4 dose** | | | |  |  | |
| --- | --- | --- | --- | --- | --- | --- | --- | --- | --- | --- | --- | --- | --- | --- | --- |
|  |  |  |  |  | **Japanese Stage 1** | | |  | **Japanese Stage 2** | | |  | **Japanese Stages 1+2** | | |
| **SNP** | **BP** | **MA** | **A2** | **Gene** | **MAF** | **N** | **OR (95% CI)** | **P** | **MAF** | **N** | **OR (95% CI)** | **P** | **OR (95% CI)** | | **P** |
| rs2965109 | 45,225,345 | T | C | CEACAM16/BCL3 | 0.434 | 1815 | 1.3 (1.12-1.51) | 4.42E-04 | 0.421 | 1803 | 1.2 (1.05-1.37) | 6.34E-03 | 1.24 (1.13-1.37) | | 1.23E-05 |
| rs7254776 | 45,227,742 | G | A | CEACAM16/BCL3 | 0.302 | 1824 | 1.27 (1.08-1.49) | 3.17E-03 | 0.301 | 1797 | 1.2 (1.05-1.39) | 9.67E-03 | 1.23 (1.11-1.37) | | 9.82E-05 |
| rs2965106 | 45,232,374 | C | T | CEACAM16/BCL3 | 0.405 | 1833 | 1.3 (1.13-1.5) | 2.43E-04 | 0.390 | 1799 | 1.3 (1.13-1.49) | 1.51E-04 | 1.3 (1.18-1.44) | | 1.33E-07 |
| rs1531517 | 45,242,173 | T | C | CEACAM16/BCL3 | 0.224 | 1829 | 1.62 (1.36-1.92) | 4.10E-08 | 0.209 | 1806 | 1.59 (1.35-1.87) | 2.41E-08 | 1.6 (1.42-1.8) | | 5.13E-15 |
| rs2927439 | 45,242,740 | C | T | CEACAM16/BCL3 | 0.387 | 1807 | 1.29 (1.11-1.49) | 7.50E-04 | 0.378 | 1785 | 1.2 (1.05-1.37) | 9.16E-03 | 1.24 (1.12-1.37) | | 2.66E-05 |
| rs8100239 | 45,253,104 | A | T | BCL3 | 0.262 | 1836 | 1.31 (1.11-1.54) | 1.36E-03 | 0.273 | 1808 | 1.29 (1.11-1.49) | 7.57E-04 | 1.29 (1.16-1.44) | | 3.37E-06 |
| rs12610605 | 45,370,838 | A | G | PVRL2 | 0.473 | 1838 | 0.63 (0.55-0.73) | 1.98E-10 | 0.481 | 1804 | 0.7 (0.61-0.79) | 7.72E-08 | 0.66 (0.6-0.73) | | 1.40E-16 |
| rs519113 | 45,376,284 | G | C | PVRL2 | 0.309 | 1838 | 2.19 (1.86-2.59) | 6.02E-21 | 0.299 | 1810 | 2.01 (1.73-2.34) | 7.65E-20 | 2.09 (1.87-2.34) | | 5.10E-39 |
| rs1160985 | 45,403,412 | T | C | TOMM40 | 0.253 | 1840 | 0.62 (0.53-0.74) | 3.75E-08 | 0.257 | 1809 | 0.65 (0.56-0.76) | 6.24E-08 | 0.64 (0.57-0.72) | | 1.27E-14 |
| rs17643262 | 45,631,816 | A | G | PPP1R37 | 0.169 | 1831 | 1.57 (1.29-1.89) | 3.81E-06 | 0.151 | 1795 | 1.46 (1.21-1.76) | 7.84E-05 | 1.51 (1.32-1.72) | | 1.40E-09 |

|  | | | **Adjusted for APOE ε4 dose** | | |  |  | |
| --- | --- | --- | --- | --- | --- | --- | --- | --- |
| **Japanese Stage 1** | |  | **Japanese Stage 2** | |  | **Japanese Stages 1+2** | | |
| **N** | **OR (95% CI)** | **P** | **N** | **OR (95% CI)** | **P** | **OR (95% CI)** | | **P** |
| 1815 | 1 (0.85-1.18) | 9.97E-01 | 1803 | 0.97 (0.85-1.12) | 7.23E-01 | 0.99 (0.89-1.1) | | 7.89E-01 |
| 1824 | 0.92 (0.77-1.1) | 3.49E-01 | 1797 | 0.91 (0.78-1.06) | 2.28E-01 | 0.91 (0.81-1.03) | | 1.27E-01 |
| 1833 | 1.03 (0.88-1.2) | 7.13E-01 | 1799 | 1.09 (0.94-1.26) | 2.51E-01 | 1.06 (0.95-1.18) | | 2.76E-01 |
| 1829 | 1.05 (0.87-1.28) | 6.12E-01 | 1806 | 1.05 (0.87-1.26) | 6.12E-01 | 1.05 (0.92-1.2) | | 4.73E-01 |
| 1807 | 0.99 (0.85-1.17) | 9.25E-01 | 1785 | 0.94 (0.81-1.09) | 4.47E-01 | 0.97 (0.87-1.08) | | 5.33E-01 |
| 1836 | 0.85 (0.71-1.03) | 9.12E-02 | 1808 | 0.93 (0.79-1.09) | 3.62E-01 | 0.89 (0.79-1.01) | | 7.17E-02 |
| 1838 | 1 (0.85-1.17) | 9.58E-01 | 1804 | 1.06 (0.91-1.23) | 4.38E-01 | 1.03 (0.92-1.15) | | 5.94E-01 |
| 1838 | 0.87 (0.7-1.09) | 2.23E-01 | 1810 | 0.92 (0.75-1.12) | 4.13E-01 | 0.9 (0.77-1.04) | | 1.54E-01 |
| 1840 | 0.91 (0.76-1.09) | 3.26E-01 | 1809 | 0.92 (0.78-1.08) | 3.07E-01 | 0.92 (0.81-1.03) | | 1.57E-01 |
| 1831 | 1.54 (1.26-1.89) | 3.08E-05 | 1795 | 1.09 (0.89-1.34) | 4.02E-01 | 1.3 (1.12-1.5) | | 3.96E-04 |

|  | |  | **APOE 3/3 subjects** | | |  |  | |
| --- | --- | --- | --- | --- | --- | --- | --- | --- |
| **Japanese Stage 1** | |  | **Japanese Stage 2** | |  | **Japanese Stages 1+2** | | |
| **N** | **OR (95% CI)** | **P** | **N** | **OR (95% CI)** | **P** | **OR (95% CI)** | | **P** |
| 1046 | 1.07 (0.87-1.31) | 5.16E-01 | 1115 | 0.93 (0.78-1.11) | 4.23E-01 | 0.99 (0.86-1.13) | | 8.53E-01 |
| 1056 | 0.95 (0.75-1.19) | 6.40E-01 | 1111 | 0.92 (0.75-1.12) | 4.01E-01 | 0.93 (0.8-1.08) | | 3.46E-01 |
| 1059 | 0.98 (0.8-1.2) | 8.54E-01 | 1112 | 1.14 (0.95-1.36) | 1.62E-01 | 1.06 (0.93-1.22) | | 3.61E-01 |
| 1053 | 1.14 (0.87-1.48) | 3.42E-01 | 1113 | 1.08 (0.84-1.38) | 5.54E-01 | 1.1 (0.92-1.32) | | 2.79E-01 |
| 1046 | 1.09 (0.89-1.34) | 4.04E-01 | 1102 | 1 (0.83-1.2) | 9.85E-01 | 1.04 (0.91-1.19) | | 5.86E-01 |
| 1058 | 0.88 (0.68-1.14) | 3.28E-01 | 1115 | 1.09 (0.88-1.33) | 4.31E-01 | 1 (0.85-1.17) | | 9.96E-01 |
| 1062 | 1.17 (0.96-1.42) | 1.13E-01 | 1113 | 0.96 (0.8-1.14) | 6.32E-01 | 1.05 (0.92-1.19) | | 4.75E-01 |
| 1061 | 0.9 (0.69-1.18) | 4.50E-01 | 1118 | 1 (0.79-1.27) | 9.98E-01 | 0.96 (0.8-1.14) | | 6.14E-01 |
| 1063 | 1.13 (0.91-1.39) | 2.66E-01 | 1115 | 1 (0.82-1.21) | 9.63E-01 | 1.05 (0.91-1.22) | | 4.74E-01 |
| 1057 | 1.39 (1.08-1.8) | 1.09E-02 | 1115 | 1.16 (0.89-1.51) | 2.78E-01 | 1.27 (1.06-1.53) | | 9.79E-03 |

|  | |  | **APOE 3/4 subjects** | | | |  |  | |
| --- | --- | --- | --- | --- | --- | --- | --- | --- | --- |
| **Japanese Stage 1** | |  | **Japanese Stage 2** | |  | **Japanese Stages 1+2** | | | |
| **N** | **OR (95% CI)** | **P** | **N** | **OR (95% CI)** | **P** | **OR (95% CI)** | | | **P** |
| 558 | 1.04 (0.76-1.43) | 7.85E-01 | 468 | 1.14 (0.85-1.52) | 3.82E-01 | 1.09 (0.89-1.35) | | | 4.07E-01 |
| 556 | 0.88 (0.64-1.2) | 4.12E-01 | 468 | 0.96 (0.71-1.29) | 7.84E-01 | 0.92 (0.74-1.14) | | | 4.44E-01 |
| 561 | 1.11 (0.83-1.48) | 4.78E-01 | 466 | 1.01 (0.75-1.34) | 9.71E-01 | 1.06 (0.86-1.3) | | | 5.99E-01 |
| 562 | 0.89 (0.65-1.23) | 4.79E-01 | 471 | 0.92 (0.67-1.27) | 6.22E-01 | 0.91 (0.72-1.14) | | | 3.96E-01 |
| 550 | 0.9 (0.66-1.23) | 5.11E-01 | 464 | 0.86 (0.64-1.16) | 3.22E-01 | 0.88 (0.71-1.09) | | | 2.42E-01 |
| 564 | 0.81 (0.59-1.12) | 2.03E-01 | 471 | 0.72 (0.52-0.98) | 3.88E-02 | 0.76 (0.61-0.96) | | | 1.82E-02 |
| 562 | 1.31 (0.9-1.9) | 1.63E-01 | 470 | 0.97 (0.68-1.39) | 8.86E-01 | 1.12 (0.87-1.45) | | | 3.91E-01 |
| 563 | 1.55 (0.98-2.44) | 5.94E-02 | 470 | 1.39 (0.91-2.12) | 1.27E-01 | 1.46 (1.07-1.99) | | | 1.64E-02 |
| 563 | 0.66 (0.42-1.03) | 6.63E-02 | 471 | 1.1 (0.7-1.74) | 6.78E-01 | 0.85 (0.62-1.16) | | | 3.07E-01 |
| 562 | 2.24 (1.44-3.48) | 3.50E-04 | 463 | 1.24 (0.84-1.81) | 2.75E-01 | 1.59 (1.19-2.12) | | | 1.57E-03 |
