# Supplementary material for: SORL1 Is Genetically Associated with Late-Onset Alzheimer’s Disease in Japanese, Koreans and Caucasians
Source: PLoS One. 2013 Apr 2;8(4):e58618. doi: 10.1371/journal.pone.0058618 (PMC3614978; doi:10.1371/journal.pone.0058618)
Supplement: Table S3 — Association results for SORL1 SNPs genotyped in the Japanese replication sample. (DOCX) [file pone.0058618.s008.docx]

**Supplementary Table 3**. Results for SORL1 SNPs genotyped in the Japanese replication sample.

|  |  |  |  | **Japanese Stage 1** | |  | **Japanese Stage 2** | |  | **Japanese Stages 1+2** | |
| --- | --- | --- | --- | --- | --- | --- | --- | --- | --- | --- | --- |
| **SNP** | **BP** | **A1** | **A2** | **MAF** | **OR (95% CI)** | **P** | **MAF** | **OR (95% CI)** | **P** | **OR (95% CI)** | **P** |
| rs4598682 | 121,375,951 | G | A | 0.23 | 0.68 (0.57-0.81) | 2.25E-05 | 0.22 | 0.83 (0.68-1.00) | 5.27E-02 | 0.75 (0.66-0.85) | 9.51E-06 |
| rs3781834 | 121,445,940 | G | A | 0.23 | 0.71 (0.60-0.85) | 1.58E-04 | 0.23 | 0.77 (0.66-0.90) | 1.09E-03 | 0.74 (0.66-0.84) | 7.33E-07 |
| rs2282647 | 121,461,593 | C | G | 0.22 | 0.69 (0.58-0.83) | 6.12E-05 | 0.23 | 1.34 (1.14-1.57) | 4.63E-04 | 1.00 (0.88-1.12) | 9.37E-01 |
| rs17125523 | 121,474,239 | G | A | 0.25 | 0.71 (0.60-0.84) | 9.05E-05 | 0.26 | 0.82 (0.70-0.95) | 9.39E-03 | 0.77 (0.68-0.86) | 5.51E-06 |
| rs3737529 | 121,477,816 | T | C | 0.25 | 0.70 (0.59-0.84) | 6.30E-05 | 0.25 | 0.82 (0.70-0.95) | 9.22E-03 | 0.77 (0.68-0.86) | 4.14E-06 |
